# The saponin monophosphoryl lipid A nanoparticle adjuvant induces dose-dependent HIV vaccine responses in nonhuman primates

**DOI:** 10.1172/JCI185292

**Published:** 2025-03-04

**Authors:** Parham Ramezani-Rad, Ester Marina-Zárate, Laura Maiorino, Amber Myers, Katarzyna Kaczmarek Michaels, Ivan S. Pires, Nathaniel I. Bloom, Mariane B. Melo, Ashley A. Lemnios, Paul G. Lopez, Christopher A. Cottrell, Iszac Burton, Bettina Groschel, Arpan Pradhan, Gabriela Stiegler, Magdolna Budai, Daniel Kumar, Sam Pallerla, Eddy Sayeed, Sangeetha L. Sagar, Sudhir Pai Kasturi, Koen K.A. Van Rompay, Lars Hangartner, Andreas Wagner, Dennis R. Burton, William R. Schief, Shane Crotty, Darrell J. Irvine

**Affiliations:** 1 Center for Vaccine Innovation, La Jolla Institute for Immunology, La Jolla, California, USA.; 2 Consortium for HIV/AIDS Vaccine Development (CHAVD), The Scripps Research Institute, La Jolla, California, USA.; 3 Department of Biological Engineering, Massachusetts Institute of Technology, Cambridge, Massachusetts, USA.; 4 Department of Immunology and Microbiology, The Scripps Research Institute, La Jolla, California, USA.; 5 Emory National Primate Research Center and Emory Vaccine Center, Emory University School of Medicine, Atlanta, Georgia, USA.; 6 Polymun Scientific Immunbiologische Forschung GmbH, Klosterneuburg, Austria.; 7 The International AIDS Vaccine Initiative Inc. (IAVI), New York, New York, USA.; 8 California National Primate Research Center and; 9 Department of Pathology, Microbiology, and Immunology, UCD, Davis, California, USA.; 10 IAVI Neutralizing Antibody Center, The Scripps Research Institute, La Jolla, California, USA.; 11 Department of Medicine, Division of Infectious Diseases and Global Public Health, UCSD, La Jolla, California, USA.; 12 Howard Hughes Medical Institute, Chevy Chase, Maryland, USA.

**Keywords:** AIDS/HIV, Immunology, AIDS vaccine, Adaptive immunity

## Abstract

Induction of durable protective immune responses is the main goal of prophylactic vaccines, and adjuvants play a role as drivers of such responses. Despite advances in vaccine strategies, development of a safe and effective HIV vaccine remains a significant challenge. Use of an appropriate adjuvant is crucial to the success of HIV vaccines. Here we assessed the saponin/MPLA nanoparticle (SMNP) adjuvant with an HIV envelope (Env) trimer, evaluating the safety and effect of multiple variables — including adjuvant dose (16-fold dose range), immunization route, and adjuvant composition — on the establishment of Env-specific memory T and B cell (T_Mem_ and B_Mem_) responses and long-lived plasma cells in nonhuman primates (NHPs). Robust B_Mem_ were detected in all groups, but a 6-fold increase was observed in the highest- versus the lowest-SMNP-dose group. Similarly, stronger vaccine responses were induced by the highest SMNP dose in CD40L^+^OX40^+^ CD4^+^ T_Mem_ (11-fold), IFN-γ^+^ CD4^+^ T_Mem_ (15-fold), IL21^+^ CD4^+^ T_Mem_ (9-fold), circulating T follicular helper cells (T_FH_; 3.6-fold), BM plasma cells (7-fold), and binding IgG (1.3-fold). Substantial tier 2 neutralizing antibodies were only observed in the higher-SMNP-dose groups. These investigations highlight the dose-dependent potency of SMNP and its relevance for human use and next-generation vaccines.

## Abstract 2

The saponin monophosphoryl lipid A nanoparticle adjuvant enhances adaptive immune responses against HIV in a dose-dependent manner in non-human primates.

## Introduction

Adjuvants are substances that promote activation of the innate immune system to induce adaptive immune responses (1–3). They can be added to vaccines to help antigens, particularly those that are weakly immunogenic, trigger protective immune responses. An ideal adjuvant potently facilitates broad and durable humoral and cellular adaptive immunity without triggering intolerable innate immune reactogenicity. Diverse adaptive immune responses to a vaccine may prevent or alleviate life-threating illness from exposures to the same and related pathogens. Within the adaptive immune system, T and B cells mediate targeted effector functions. Vaccine-specific CD4^+^ and CD8^+^ T cells are characterized by helper and cytotoxic activity, respectively. Both CD4^+^ and CD8^+^ T cells can develop into long-lived memory T cells (T_Mem_) capable of rapidly reactivating upon antigen reencounter. Specific CD4^+^ helper T cells, known as T follicular helper cells (T_FH_), are instrumental in activating B cells and facilitating B cell proliferation and differentiation during the immune reaction (4). B cells are induced to generate protective antibodies and typically affinity mature their antibodies in germinal centers (GCs). Major outputs of GC responses are memory B cells (B_Mem_) and terminally differentiated plasma cells (B_PC_). B_Mem_ serve as a resting memory population capable of reactivating upon antigen reencounter. B_Mem_ and B_PC_, together with T_Mem_, constitute immune memory, which may have differing durability depending on the vaccine.

Emerging adjuvants are developed to induce only necessary immune effector functions (2). For example, adjuvants have been developed that are ligands for TLRs, innate immune receptors in host cells that recognize pathogen-associated molecular patterns (PAMPs). The TLR4 agonist monophosphoryl lipid A (MPLA), a derivative of LPS, is a component of two adjuvant systems, AS01 and AS04, which are used in licensed vaccines produced by GlaxoSmithKline. MPLA has been shown to enhance immune responses, likely via stimulation of dendritic cells (5). Other licensed and investigated TLR agonists include TLR9 agonists (CpG DNA) and TLR7/TLR8 agonists (3M-052), respectively, which are capable of enhancing humoral immune responses as vaccine adjuvants (6, 7).

Another important class of vaccine adjuvant compounds are saponins, triterpene glycosides originally isolated from the Chilean soapbark tree *Quillaja saponaria*. Saponin-containing adjuvants more potently induce B cell responses compared with aluminum salts (8–10) and are employed in licensed vaccines against malaria, varicella zoster virus, and SARS-CoV-2 (11–13). We recently described an adjuvant termed SMNP, which has a cage-like nanoparticle structure characteristic of saponin-based immune-stimulating complexes (ISCOMs) (14, 15); and contains both saponin and MPLA, which exhibited high potency in mice and nonhuman primates (NHPs) (8). SMNP is particularly promising for unmet vaccine needs such as HIV. No effective HIV vaccines are currently available, and conventional vaccine strategies have not induced successful protection against diverse HIV strains (16, 17). Advances have been made in immunogen design and vaccine regimens, including germline targeting (18–21), but all prophylactic HIV vaccine strategies depend on appropriate adjuvants to drive recruitment of rare B cells capable of targeting neutralizing epitopes on HIV to produce effective GC responses. GCs provide a highly specialized environment for B cells to affinity mature antibodies against difficult-to-target epitopes on HIV. Notably, SMNP increases lymph flow and antigen acquisition in draining lymph nodes (LNs) (8), which can augment GC responses and facilitate improved neutralizing responses (18, 22). Studies in mice provide valuable early immunological assessments for immunogens and adjuvants. However, NHPs offer a superior model for evaluating immune responses to HIV vaccines and serve as more accurate late preclinical models for humans due to their susceptibility to similar or identical pathogens (23). Immune memory, including durable antibody responses, is critical for the long-term benefit of prophylactic vaccines; however, direct measurement of the effects of adjuvant choice and dose on B and T cell memory development in humans or NHPs is less common than evaluation of antibody responses, leaving significant knowledge gaps.

Accepting SMNP as a promising vaccine adjuvant, we investigated several as-yet-unexplored dimensions of its adjuvant activity. Saponins employed in vaccines are most often natural products. Preclinical studies to date with SMNP have employed a relatively crude saponin extract, termed Quil-A, that contains a mixture of many saponin fractions (24). By contrast, for clinical adjuvant products, a highly purified saponin isolate termed QS-21 is preferred, which is composed of just 2 isomers of a single saponin species. QS-21 is immunologically potent and has been employed in the approved liposomal adjuvant AS01_B_ in the Shingrix vaccine (for the prevention of shingles caused by varicella zoster) (12). The approved ISCOM-class adjuvant Matrix-M in the Novavax COVID-19 vaccine (for the prevention of COVID-19 caused by SARS-CoV-2) employs 2 saponin fractions (fraction-A and -C), but displays a nanoparticle structure similar to that of SMNP (25). AS01_B_ has been shown to induce strong humoral and cellular immune responses (26). However, doses of AS01_B_ have been limited to 50 μg for human use, likely due to its strong reactogenicity. Additionally, the liposomal nature of AS01_B_ requires special storage and handling conditions. For example, AS01_B_ needs to be handled carefully to maintain structural integration and cannot be stored mixed with the antigen prior to immediate administration, in contrast to the ISCOM-based saponin adjuvant Matrix-M, which is supplied together with the antigen. Consequently, the ISCOM structure of SMNP may offer favorable stability during storage and handling, which could be advantageous for ease of vaccine administration. However, a better understanding of the effect of SMNP on immune responses is needed. Thus, key questions include: (i) How does the saponin source affect the strength and durability of immune responses? (ii) How do SMNP formulations compare with those of a related licensed saponin-based adjuvant (AS01_B_)? (iii) What are the effects of dose or route of SMNP administration on immune responses and reactogenicity? More broadly, it is of high value to directly measure the effect of adjuvant choice and dose on B and T cell memory development in humans or NHPs. These aspects were investigated in this study to provide a broader understanding of saponin-based adjuvants in primates.

## Results

### QS-21 SMNP tested with an HIV vaccine in NHPs.

**Figure 1:**
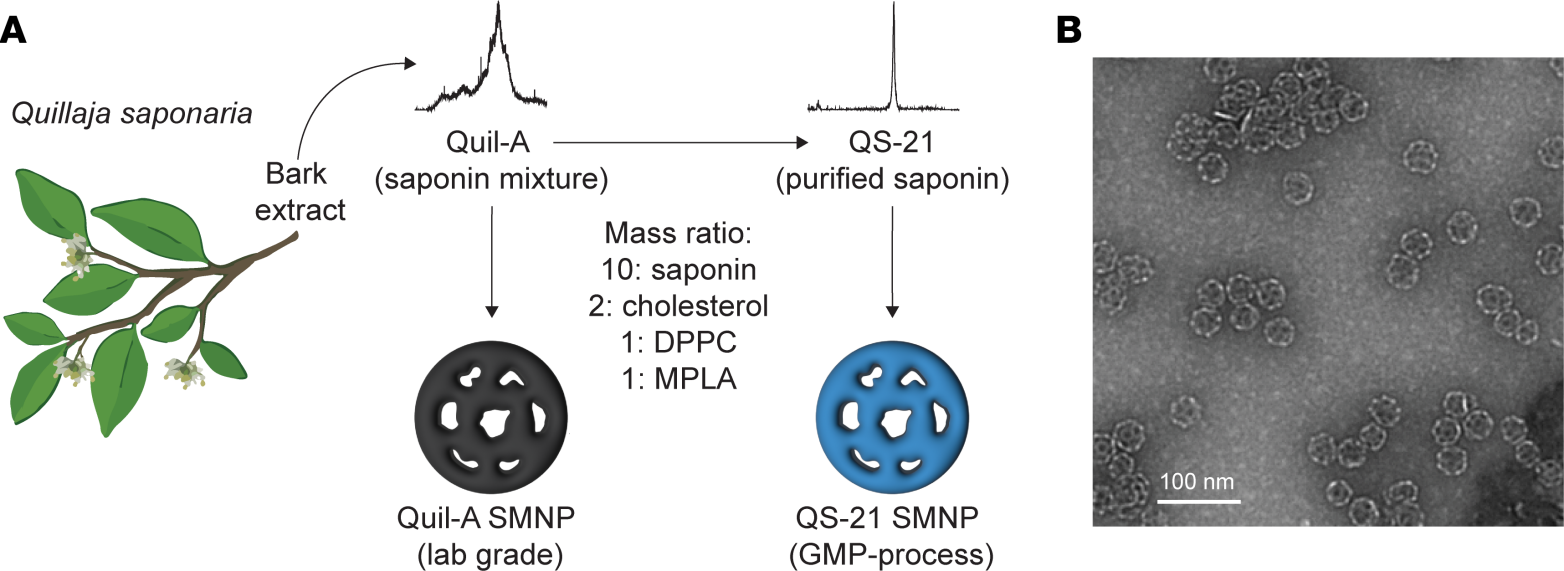
Saponin-based nanoparticles. (**A**) Formulation of SMNP with Quil-A (laboratory grade) or QS-21 (GMP-process). (**B**) Representative TEM image of QS-21 SMNP. Scale bar: 100 nm.

We previously characterized and tested SMNP prepared using Quil-A saponin (laboratory-grade SMNP) in mice and NHPs (8). Clinical-grade current good manufacturing practice (cGMP) SMNP synthesized using QS-21 was recently prepared for a human clinical trial (HVTN144, ClinicalTrials.gov NCT06033209) using a scalable manufacturing process based on tangential flow filtration (27). We collected QS-21 SMNP produced using the same process during an engineering run for this manufacturing scale-up for the present study to assess whether scale-up manufacturing methods or the saponin source affected responses in NHPs. Further, we aimed to carry out immunizations in rhesus macaques mirroring the planned clinical dose escalation to enable comparisons between the NHP and human responses to this adjuvant. For both laboratory-grade SMNP prepared with Quil-A saponin and the GMP-process SMNP prepared with QS-21 saponin, the mass ratios of saponin, cholesterol, dipalmitoylphosphatidylcholine (DPPC), and MPLA used in the adjuvant synthesis were the same (Figure 1A) (8). Characterization of the QS-21 SMNP showed that it had the expected cage-like nanoparticle morphology (Figure 1B), with particle size and composition mirroring those of laboratory-grade preparations used in our previous work (Supplemental Table 1; supplemental material available online with this article; https://doi.org/10.1172/JCI185292DS1) (8).

**Figure 2:**
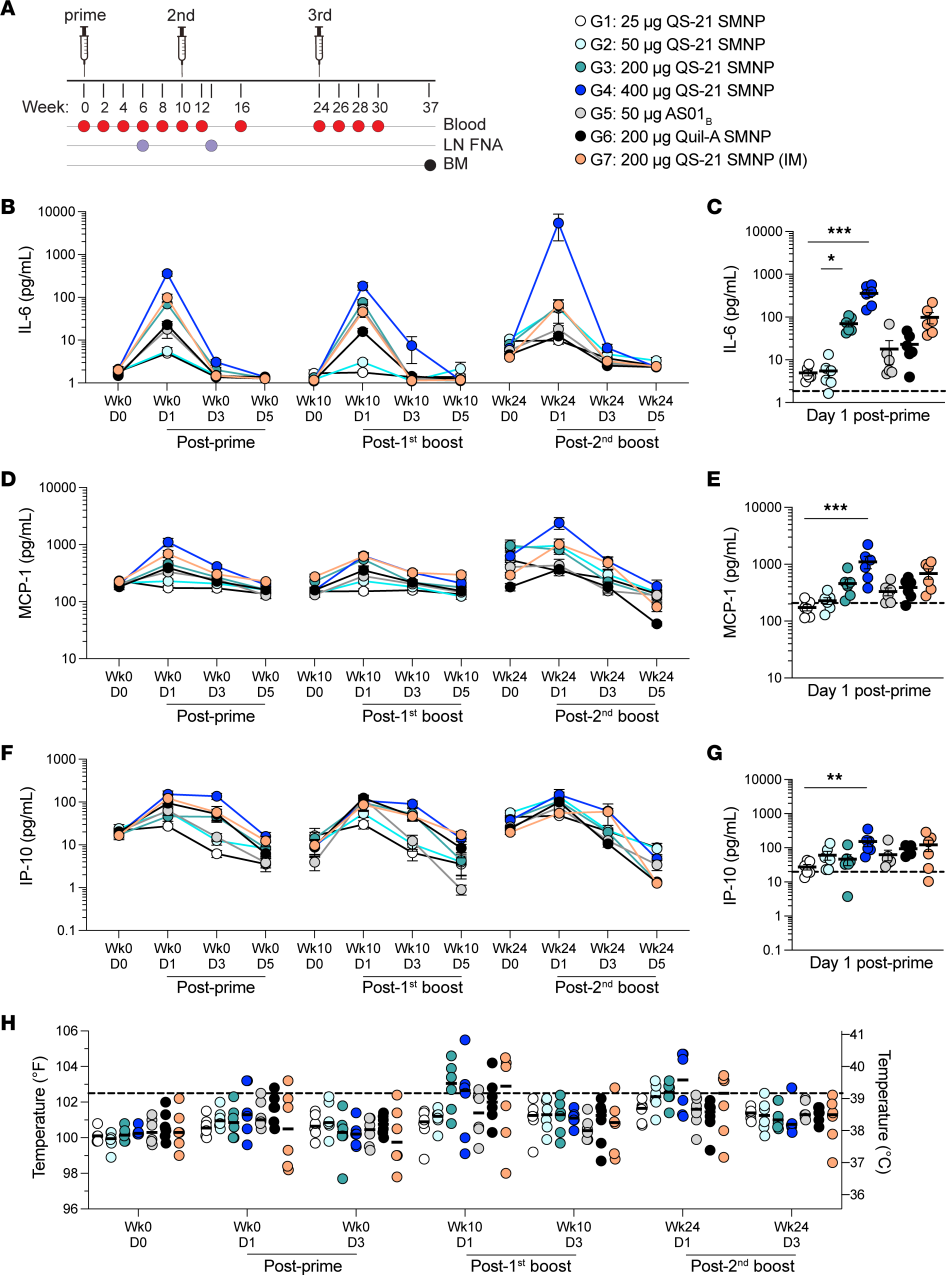
Increased plasma cytokine levels are detected with higher QS-21 SMNP doses. (**A**) Overview of immunizations and sample collection schedule for NHPs. (**B**–**G**) Mean plasma cytokine concentration at baseline (week 0, day 0 [Wk0 D0]) and days 1–5 (D1–D5) following immunizations: IL-6 (**B** and **C**), MCP-1 (**D** and **E**), and IP-10 (**F** and **G**). (**H**) Measurement of rectal body temperature in degrees Fahrenheit (left *y* axis) and Celsius (right *y* axis) at baseline (D0) or D1–3 following immunizations. Dotted line is set at 102.5°F/39.2°C and represents the temperature threshold for fever in NHPs. Bars represent median per group. In **B**–**G**, error bars represent mean with SEM. Statistical significance was assessed using the Kruskal-Wallis test, followed by Dunn’s multiple-comparison test. *P ≤ 0.05, **P < 0.01, and ***P < 0.001. In **C**, **E**, and **G**, the dotted line represents the mean baseline (Wk0 D0) response of all groups. All data represent *n* = 6 animals/group.

A total of 42 rhesus macaques were divided in 7 different groups with 6 animals per group. Different QS-21 SMNP doses (25 μg, 50 μg, 200 μg, 400 μg) were evaluated in groups 1–4. The groups in which GMP-process SMNP was employed were compared with the groups receiving laboratory-grade Quil-A SMNP (group 6) and AS01_B_ (group 5). Route of vaccine administration was also examined for QS-21 SMNP (group 7). All groups received 100 μg HIV envelope (Env) trimer BG505 MD39 protein coadministered with the adjuvant (28). A total of 3 doses of protein plus adjuvant were administered at weeks 0, 10, and 24, unilaterally in the thighs for the s.c. groups (groups 1–6) or intramuscularly (i.m.) in the deltoid group (group 7) (Figure 2A).

After each immunization, reactogenicity and levels of cytokines/chemokines in plasma were evaluated. Plasma levels of IL-6, MCP-1 (CCL2), and IP-10 (CXCL10) peaked at day 1 and declined to baseline levels by day 3 or 5 (Figure 2, B–G). These cytokines are known to be involved in inflammatory responses and can promote the activity of adaptive immune responses, as well as indicating systemic reactogenicity. Somewhat surprisingly, although the doses spanned a 16-fold range, a plateau in SMNP activity was not reached. At day 1 after priming, 400 μg QS-21 SMNP (group 4) led to the highest IL-6 levels, which were significantly greater than those resulting from 25 μg QS-21 SMNP (group 1 vs. 4, *P* = 0.0001; Figure 2C). Similarly, 400 μg QS-21 SMNP (group 4) induced higher MCP-1 levels than 25 μg QS-21 SMNP (group 1 vs. 4, *P* = 0.0002; Figure 2E), and 400 μg QS-21 SMNP also induced greater IP-10 levels than 25 μg QS-21 SMNP (group 1 vs. 4, *P* = 0.0058; Figure 2G). Plasma cytokine responses to 50 μg AS01_B_ (group 5) were comparable to the those with the equivalent 50 μg QS-21 SMNP dose (group 2). Levels of IL-8, IL-10, IL-1β, IL-12p40, IL-17A, IFN-β, IL-23, TNF, IFN-γ, and GM-CSF did not change from baseline (data not shown). Overall, plasma cytokine concentrations were associated with QS-21 SMNP dose, but the duration of cytokine elevation did not appear to change with adjuvant dose.

Transient systemic reactogenicity (a temperature of at least 102.5°F/39.2°C was considered a fever) was apparent in some animals, particularly with higher SMNP doses (≥200 μg) (Figure 2H). After the first immunization, no substantial temperature changes were observed (Supplemental Figure 1A). At 1 day after the second immunization, fever-level temperatures were observed in a number of animals, with significant temperature elevations above baseline with 200 μg QS-21 SMNP (group 3, *P* = 0.0007; Supplemental Figure 1B). Similar outcomes were seen after the third immunization, with significant temperature elevations with 25 μg, 50 μg, 200 μg, and 400 μg QS-21 SMNP (groups 1–4, *P* < 0.01; Supplemental Figure 1C). These temperature excursions returned to baseline by day 3 in all groups. No major difference was observed between s.c. or i.m. routes of administration (group 3 vs. 7). Temperature changes with 50 μg AS01_B_ (group 5) were overall comparable to those with the equivalent QS-21 SMNP dose (group 2). Transient redness, mild rashes, and swelling were observed for 1–3 days after immunization in some animals (data not shown), but no unexpected or concerning local reactogenicity was observed.

### Dose-dependent effects of SMNP on antigen-binding memory B_Mem_.

**Figure 3:**
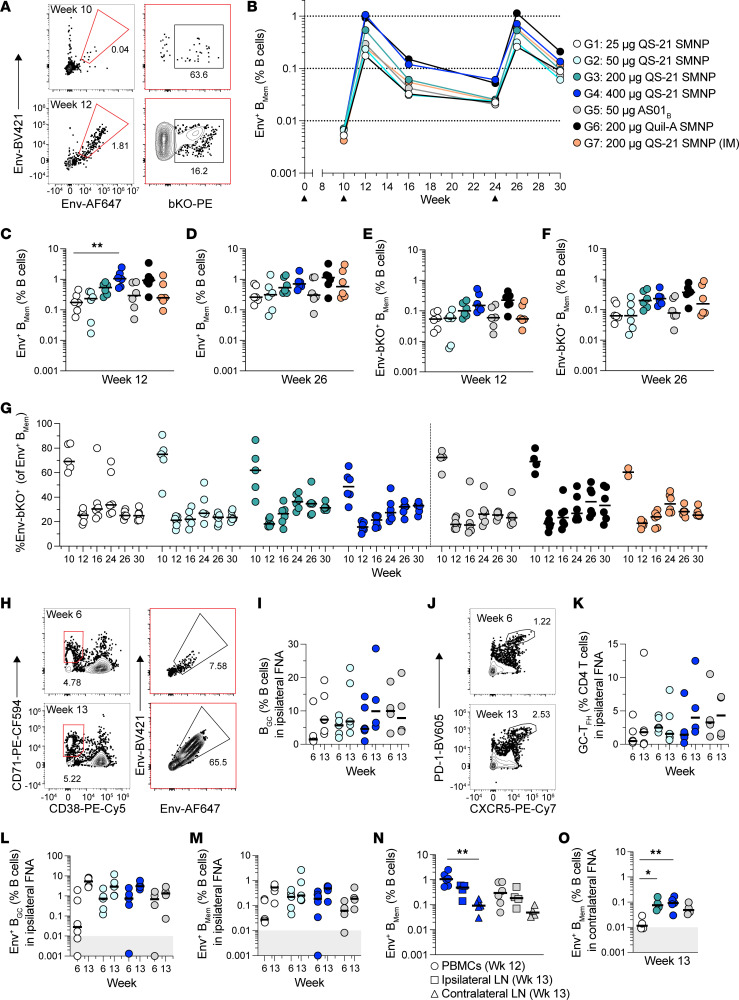
Robust longitudinal Env-binding B_Mem_ are detected with high QS-21 SMNP doses. (**A**) Representative flow cytometry plots of Env-binding B_Mem_ (Env-BV421^+^Env-AF647^+^) and Env bKO-binding B_Mem_ (bKO-PE^+^) in PBMCs at weeks 10 and 12. (**B**) Env-binding B_Mem_ as a percentage of total B cells (medians per group) in PBMCs at different time points after immunization. Black triangles indicate times of immunizations. The LOD for Env-binding B_Mem_ was 0.0006%. (**C** and **D**) Frequency of Env-binding B_Mem_ in PBMCs at week 12 (**C**) and week 26 (**D**). (**E** and **F**) Frequency of bKO-binding B_Mem_ in PBMCs at week 12 (**E**) and week 26 (**F**). (**G**) bKO-binding B_Mem_ as a percentage of Env-binding B_Mem_. (**H**) Representative flow cytometry plots of total B_GC_ (CD71^+^CD38^–^) and Env-binding B_GC_ (Env-BV421^+^Env-AF647^+^) in ipsilateral LN FNAs at weeks 6 and 13. (**I**) Frequency of total B_GC_ in ipsilateral LN FNAs at weeks 6 and 13. (**J**) Representative flow cytometry plots of GC-T_FH_ (PD-1^hi^CXCR5^+^) in ipsilateral LN FNAs at weeks 6 and 13. (**K**) Frequency of GC-T_FH_ in ipsilateral LN FNAs at weeks 6 and 13. (**L**) Frequency of Env-binding B_GC_ in ipsilateral LN FNAs at weeks 6 and 13. (**M**) Frequency of Env-binding B_Mem_ in ipsilateral LN FNAs at weeks 6 and 13. (**N**) Frequency of Env-binding B_Mem_ compared across 3 different tissues (PBMCs, ipsilateral LN, and contralateral LN). (**O**) Frequency of Env-binding B_Mem_ in contralateral LN FNAs at week 13. Bars represent the median per group. Gray area in **L**, **M**, and **O** indicates the LOD. Statistical significance was assessed using the Kruskal-Wallis test, followed by Dunn’s multiple-comparison test. *P ≤ 0.05 and **P < 0.01. All data represent *n* = 6 animals/group unless the exclusion criteria described in Methods apply.

Env-binding B_Mem_ were analyzed longitudinally by flow cytometry at different time points after priming (week 10) as well as after boost 1 (weeks 12, 16, and 24) and boost 2 (weeks 26 and 30). At week 10, Env-binding B_Mem_ were detectable in all groups before a booster immunization (Figure 3, A and B). Two weeks after the first boost (week 12), the Env-binding B_Mem_ expanded substantially in all groups (Figure 3, B and C). The highest QS-21 SMNP dose (400 μg; group 4) and 200 μg Quil-A SMNP (group 6) induced the highest frequencies of Env-binding B_Mem_ responses, with median Env-binding B_Mem_ representing approximately 1% of total B cells (Figure 3C). Env-binding B_Mem_ responses at week 12 were positively associated with QS-21 SMNP dose, with 159-fold B_Mem_ expansion in the 400 μg QS-21 SMNP group (week 12 vs. 10; Figure 3, B and C). Responses to 50 μg AS01_B_ (group 5) were comparable to those with the corresponding 50 μg QS-21 SMNP dose (group 2). Additionally, the 200 μg Quil-A SMNP dose group (group 6) had Env-binding B_Mem_ responses similar to those of the equivalent QS-21 SMNP dose group (group 3). Delivery of 200 μg QS-21 SMNP s.c. (group 3) or i.m. (group 7) elicited robust and comparable Env-binding B_Mem_ responses. The Env-binding B_Mem_ frequency of all groups was maintained longitudinally after the boost despite a decline observed with time in all groups (Figure 3B and Supplemental Figure 2B). The consistency of the decline of Env-binding B_Mem_ indicated that differentiated B_Mem_ with qualitatively similar durability were generated across the study groups (Supplemental Figure 2, D and E), but they differed in magnitude associated with the adjuvant dose. The 400 μg QS-21 SMNP (group 4) Env-binding B_Mem_ half-life observed between weeks 12 and 16 was 9 days and over weeks 16–24 was 48 days. After the second boost (week 26), there was strong increase in Env-binding B_Mem_ compared with pre-boost cells (week 24) for all groups (Figure 3, B and D). These Env-binding B_Mem_ responses declined by week 30, showing a pattern of decline comparable to that observed after boost 1. Overall, Env-binding B_Mem_ were induced in all QS-21 SMNP dose groups after priming and substantially increased after boosts. The B_Mem_ half-lives suggest a transient expansion of circulating B_Mem_ shortly after booster immunization, with the cells remaining 6 weeks later exhibiting a longer half-life.

Utilization of a soluble recombinant Env protein immunogen creates the issue of an immunodominant Env base surface that is otherwise buried in the membrane of HIV (29). An Env base-KO (bKO) probe (18) was used to identify B_Mem_ specific for non-base epitopes important for HIV neutralization (Figure 3A). Consistent with total Env-binding B_Mem_, bKO-binding B_Mem_ were detectable after priming (week 10) in all groups (Supplemental Figure 2F). After boosting (weeks 12 and 26), bKO-binding B_Mem_ were strongly enhanced and associated with the QS-21 SMNP dose (Figure 3, E and F, and Supplemental Figure 2F). The proportion of bKO-binding B_Mem_ among total Env-binders was the highest at the pre-boost time point (week 10) and then dropped sharply after boosting (week 12; Figure 3G). This outcome is consistent with the strongly competitive base responses shown previously (22, 30). The proportion of bKO-binders at week 12 or later trended higher for groups receiving QS-21 or Quil-A SMNP doses of at least 200 μg (Figure 3G). The bKO-binding B_Mem_ declined after the boost but were strongly induced after the second boost (week 26) in all groups (Supplemental Figure 2F). Overall, durable bKO-binding B_Mem_ frequencies were induced in all groups, but were stronger in higher-SMNP-dose groups.

Fine needle aspiration (FNA) of LNs allows sampling of tissue-adaptive immune responses, including GC responses (30, 31). Select NHP groups were analyzed for total and Env-binding B_GC_ in ipsilateral LNs after priming (week 6) and after boosting (week 13; Figure 3, H and I). Total B_GC_ were detected at both time points with comparable frequencies (Figure 3I). Total GC T follicular helper cells (GC-T_FH_) were also comparable across groups and time points (Figure 3, J and K). Env-binding B_GC_ trended higher after boosting compared with after priming, but levels were similar in the 50 and 400 μg QS-21 SMNP dose groups at both time points (Figure 3L). Local Env-binding B_Mem_ was detectable in the draining ipsilateral LNs, and trending increases were detected after boosting compared with after priming (Figure 3M). We have previously shown that peak immune responses in PBMCs occur approximately 2 weeks after the boost and in LNs approximately 3 weeks after the boost (18, 30). The post-boost (week 13) Env-binding B_Mem_ frequencies in the ipsilateral LN were trending lower for the 400 μg QS-21 SMNP group compared with the circulating post-boost Env-binding B_Mem_ (week 12) frequencies in the blood (Figure 3N). For the 50 μg AS01_B_ group, week 13 Env-binding B_Mem_ frequencies in the ipsilateral LN were comparable with the circulating week 12 Env-binding B_Mem_ frequencies.

The unilateral nature of this study allowed analysis of the contralateral nondraining LN for Env-binding B cell responses. As expected, no Env-binding B_GC_ responses were detected in the contralateral LN (Supplemental Figure 3C). Env-binding B_Mem_ were detectable in contralateral LN after the boost, highlighting effective recirculation of B_Mem_ (Figure 3O and Supplemental Figure 3C). Env-binding B_Mem_ frequencies in contralateral LN were 8-fold higher for the 400 μg QS-21 SMNP group compared with the 25 μg QS-21 SMNP group (group 1 vs. 4, *P* = 0.0084; Figure 3O). The frequency of Env-binding B_Mem_ in the contralateral LN was considerably lower than in the ipsilateral LN for 400 μg QS-21 SMNP and for AS01_B_ (Figure 3N). Overall, the dose of QS-21 SMNP was associated with the frequency of Env-binding B_Mem_ in circulation and in LNs.

### BCR sequencing of antigen-binding memory B cells.

**Figure 4:**
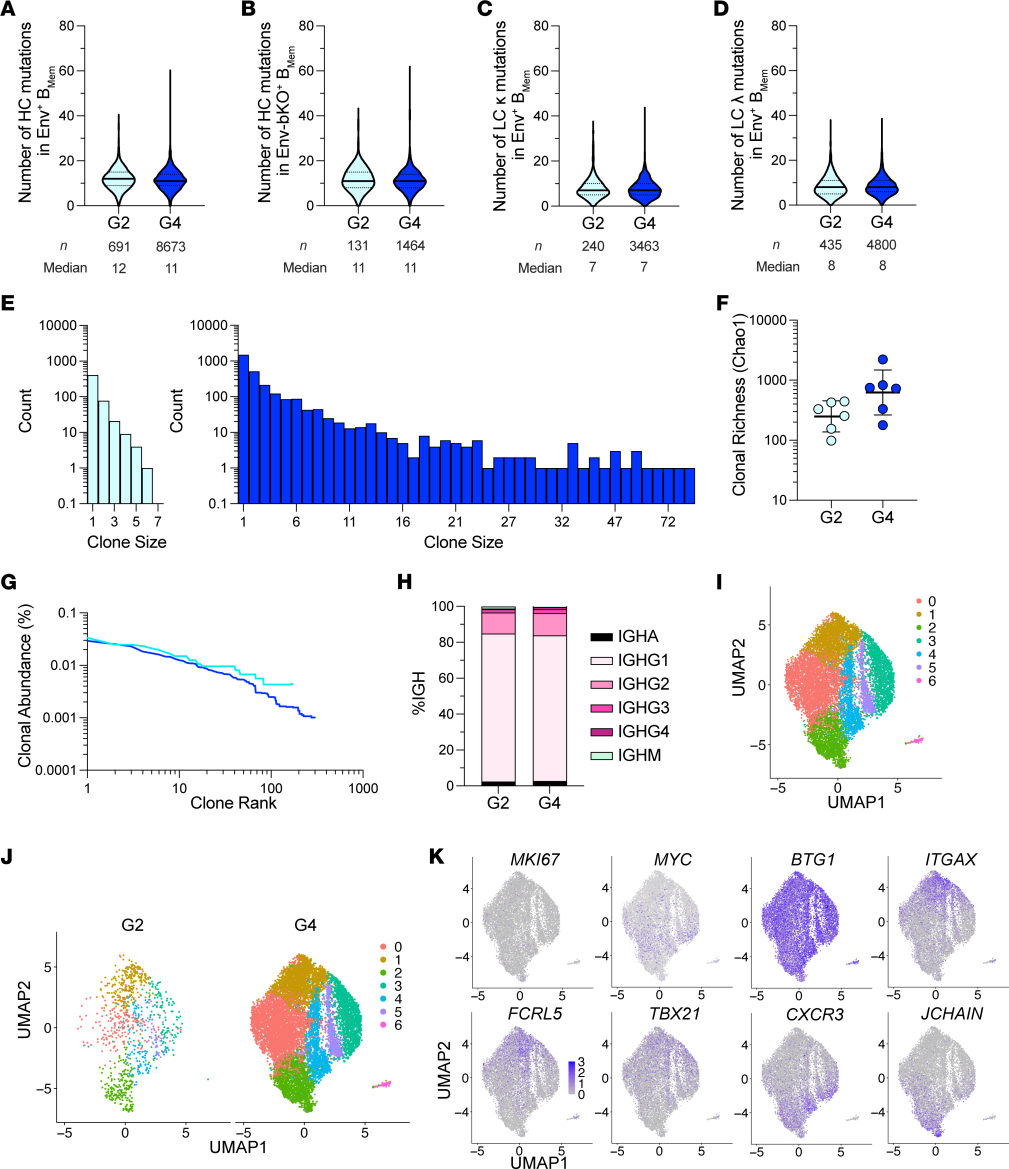
Single B cell sequencing of Env-binding B_Mem_ in low- and high-dose QS-21 SMNP groups. (**A** and **B**) Number of HC nucleotide mutations in Env-binding B_Mem_ (**A**) and bKO-binding B_Mem_ (**B**) at week 12 in PBMCs. G, group. (**C** and **D**) Number of LC κ (**C**) and LC λ (**D**) mutations in Env-binding B_Mem_ at week 12. (**E**) Distribution of clonotype sizes for groups 2 and 4. (**F**) Clonal richness (Chao1) of Env-binding B_Mem_. (**G**) Clonal abundance curve of Env-binding B_Mem_. (**H**) Proportion of Ig isotypes among Env-binding B_Mem_, shown as a percentage of total IGH. (**I**–**K**) Uniform manifold approximation and projection (UMAP) visualization of single-cell gene expression profiles identifying clusters of total Env-binding B_Mem_ at week 12 (**I**) across groups (**J**) and for specific markers (**K**). In **A**–**D**, the solid black line and dotted lines represent the median and quartiles, respectively. The error bars in **F** represent the geometric mean with geometric SD. Animals were excluded from clonal abundance curve in **G** if fewer than 50 sequences were recovered; otherwise, all data represent *n* = 6 animals/group. Statistical significance was assessed using an unpaired 2-tailed Mann-Whitney *U* test.

Flow cytometric characterization of Env-binding B_Mem_ elucidated a difference in the magnitude of B_Mem_ responses based on the dose of QS-21 SMNP. To gain further insights into the quality and diversity of the Env-binding B_Mem_ at low and high adjuvant doses, we performed single-cell BCR sequencing using week 12 PBMCs from all animals that received 50 μg or 400 μg QS-21 SMNP (groups 2 and 4). Somatic hypermutation (SHM) was similar in the 50 μg and 400 μg QS-21 SMNP groups for total Env- and bKO-binding B_Mem_ (Figure 4, A–D and Supplemental Figure 4, A–D). Regarding clonal diversity, the higher frequency of Env-binding B_Mem_ (Figure 3C) in the 400 μg group compared with the 50 μg group was reflected in a greater abundance of BCR clones recovered (Figure 4E). The clonal richness of those B_Mem_ trended higher for the higher SMNP dose (Chao1, *P* = 0.065), but the increase did not reach statistical significance (Figure 4F). Similarly, a rank abundance test showed no major differences in clonal abundance (Figure 4G).

Env-binding B_Mem_ were predominantly IgG1^+^, and overall Ig isotype profiles were comparable between the groups (Figure 4H). Single-cell transcriptomic analysis of gene expression profiles of Env-binding B_Mem_ indicated major overlap in Env-binding B_Mem_ phenotypes between the 2 SMNP doses (Figure 4, I–K, and Supplemental Figure 4E). The proportions of Env-binding B_Mem_ were also very similar for most clusters between the groups, except some variability for a few clusters (Supplemental Figure 4F). The majority of cells appeared to be resting nonproliferative, as indicated by low levels of *MKI67* and *MYC* expression and broader expression of antiproliferative *BTG1* (Figure 4K). Some atypical B_Mem_ markers, including *ITGAX* (CD11c), *FCRL5*, and *TBX21* (T-bet) were largely found in cluster 1. *CXCR3* appeared to be distinctly expressed compared with *ITGAX*. *CXCR3* was expressed in several clusters, including cluster 2, which also contained a subcluster of cells expressing *JCHAIN,* likely indicating a small population of B_PC_ (Figure 4K and Supplemental Figure 4G). Overall, the diversity of B_Mem_ clonotypes and phenotypes, as well as the quality of these cells, was similar with low and high doses of QS-21 SMNP.

### Dose-dependent effects of SMNP on antigen-specific memory T cells.

**Figure 5:**
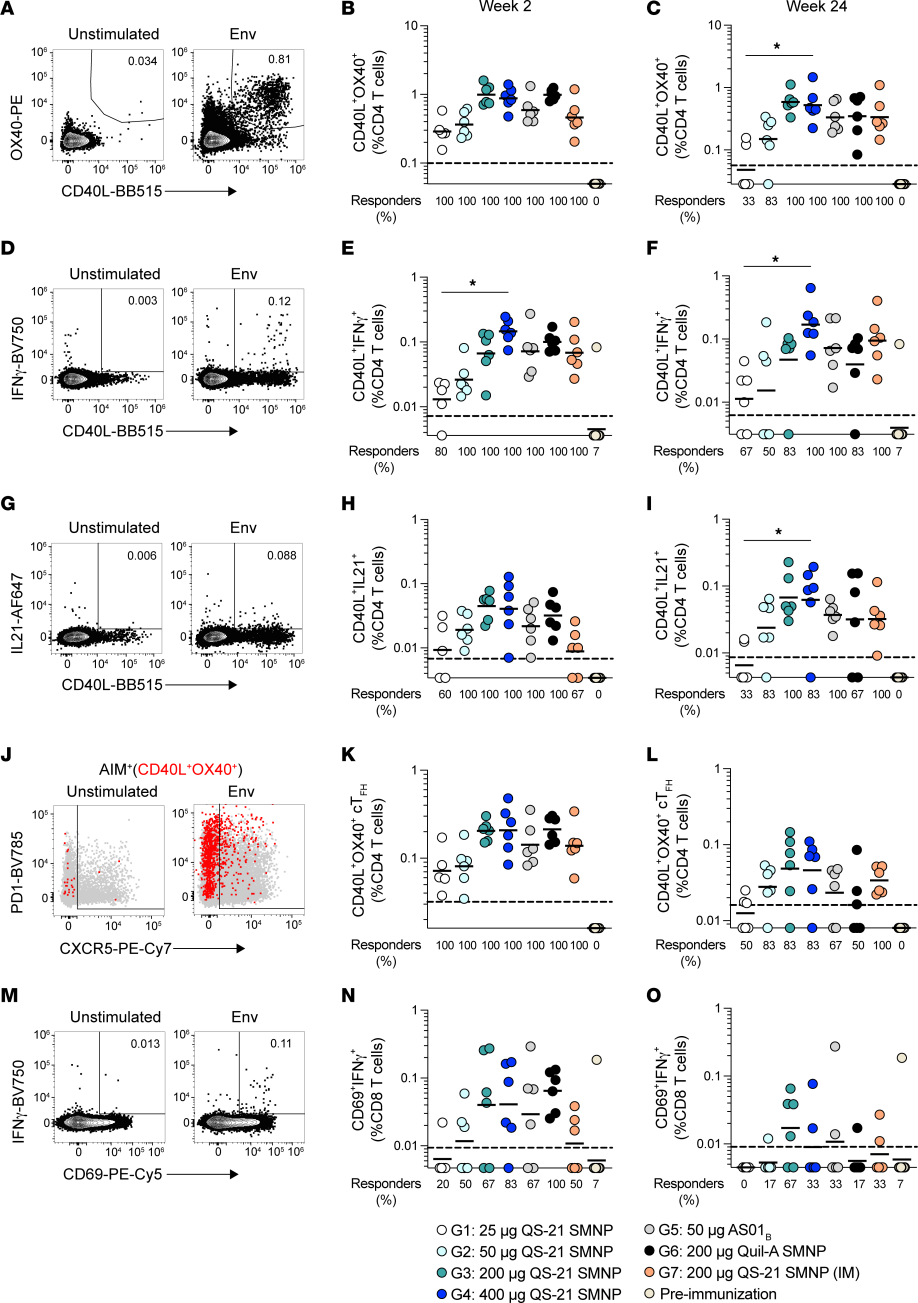
Robust Env-specific T cells are detected with all QS-21 SMNP doses after priming. (**A**) Representative flow cytometry plots of AIM^+^ (OX40^+^CD40L^+^) CD4^+^ T cells from unstimulated or Env peptide pool–stimulated samples at week 2. (**B** and **C**) Frequency of AIM^+^ (OX40^+^CD40L^+^) Env-specific CD4^+^ T cells at week 2 (**B**) and week 24 (**C**). (**D**) Representative flow cytometry plots of CD40L^+^IFN-γ^+^ CD4^+^ T cells from unstimulated or Env peptide pool–stimulated samples at week 2. (**E** and **F**) Frequency of CD40L^+^IFN-γ^+^ Env-specific CD4^+^ T cells at week 2 (**E**) and week 24 (**F**). (**G**) Representative flow cytometry plots of CD40L^+^IL-21^+^ CD4^+^ T cells from unstimulated or Env peptide pool–stimulated samples at week 2. (**H** and **I**) Frequency of CD40L^+^IL-21^+^ CD4^+^ T cells at week 2 (**H**) and week 24 (**I**). (**J**) Representative flow cytometry plots of cT_FH_ (CXCR5^+^) with AIM^+^ (OX40^+^CD40L^+^) (red) at week 2. (**K** and **L**) Frequency of AIM^+^ (OX40^+^CD40L^+^) Env-specific cT_FH_ at week 2 (**K**) and week 24 (**L**). (**M**) Representative flow cytometry plots of CD69^+^IFN-γ^+^ CD8^+^ T cells from unstimulated or Env peptide pool-stimulated samples at week 2. (**N** and **O**) Frequency of CD69^+^IFN-γ^+^ Env-specific CD8^+^ T cells at week 2 (**N**) and week 24 (**O**). Horizontal bars represent the geometric mean per group. Black dotted lines indicate the LOQ. Percent responders was calculated as the percentage of animals with levels above the LOQ. Frequencies are shown after subtraction from paired unstimulated samples. Statistical significance was assessed using the Kruskal-Wallis test, followed by Dunn’s multiple-comparison test. *P ≤ 0.05 and **P < 0.01. All data represent *n* = 6 animals per group, except for group 1, which had *n* = 5 at week 2. Preimmunization data (*n* = 14) were derived from different groups of animals.

T cell responses were compared across the study groups. Acute and memory Env-specific T cell responses were investigated after priming (week 2) and 14 weeks after boost 1 (week 24) in activation-induced marker (AIM) and intracellular cytokine staining (ICS) flow cytometric assays. Env-specific CD4^+^ T cell responses were induced in all animals of all groups after priming, as detected by AIM (AIM^+^ = OX40^+^CD40L^+^; Figure 5, A and B, gated as per Supplemental Figure 5A). The highest dose of QS-21 SMNP (400 μg, group 4) induced a marked increase of AIM^+^ Env-specific CD4^+^ T cells compared with the lowest dose after priming (Figure 5B). After the boost, memory Env-specific CD4^+^ T cells were well maintained with the 2 highest QS-21 SMNP doses (groups 3 and 4), but were substantially lower for the 25 and 50 μg QS-21 SMNP groups 1 and 2, with an 11-fold difference between lowest and highest QS-21 SMNP groups (group 1 vs. 4, *P* = 0.013; Figure 5C).

In response to QS-21 SMNP, T cells also showed dose-dependent differences in IFN-γ production at acute (week 2) and memory (week 24) time points (group 1 vs. 4 at week 2, 11-fold, *P* = 0.01; group 1 vs. 4 at week 24, 15-fold, *P* = 0.015; Figure 5, D–F, gated as per Supplemental Figure 5A). Similar trends were observed for Env-specific CD4^+^ T cells producing IL-2 or TNF (Supplemental Figure 5, F–I). Significant differences in TNF were observed for the lowest– and highest–QS-21 SMNP groups (group 1 vs. 4 at week 2, 8-fold, *P* = 0.0067; group 1 vs. 4 at week 24, 8-fold, *P* = 0.036; Supplemental Figure 5, H and I). AS01_B_ (group 5) induced higher-trending levels of acute CD4^+^ T cells producing IFN-γ or IL-2 compared with QS-21 SMNP (Figure 5E and Supplemental Figure 5F). IL-21–producing Env-specific CD4^+^ T cells increased with higher QS-21 SMNP doses, with marked increases in IL-21^+^ Env-specific CD4^+^ T cells in group 4 compared with group 1 in the acute phase (Figure 5, G and H) and 9-fold more IL-21^+^ memory CD4^+^ T cells at week 24 (*P* = 0.019; Figure 5I).

Compared with s.c. immunization, i.m. immunization induced noticeably fewer acute and memory AIM^+^ Env-specific CD4^+^ T cells (Figure 5, B and C) and IL-21–producing Env-specific CD4^+^ T cells (Figure 5, H and I).

Antigen-specific circulating T_FH_ (cT_FH_) are one indicator of antigen-specific GC-T_FH_ in lymphoid organs (32–35). AS01_B_ in combination with a subunit recombinant protein immunogen has been shown to elicit higher cT_FH_ compared with a viral vector vaccination regimen (36). Acute Env-specific cT_FH_ were induced in all groups after priming, but levels were substantially higher in animals receiving 200–400 μg QS-21 SMNP or 200 μg Quil-A SMNP (Figure 5, J and K). By week 24, Env-specific memory cT_FH_ remained detectable in the majority of animals receiving 50–400 μg QS-21 SMNP (Figure 5L). Overall, Env-specific CD4^+^ T cells were primed in all groups, and memory CD4^+^ T cell frequencies were greater at higher QS-21 SMNP doses.

CD8^+^ T cell responses are not reported for most human or NHP adjuvanted protein vaccines. Nevertheless, multiple reports exist of CD8^+^ T cell responses in humans and NHPs in the context of saponin-based adjuvants, including SMNP (22, 37, 38). Acute Env-specific CD8^+^ T cell (CD69^+^IFN-γ^+^) responses were elicited in some animals at week 2, with more responders at a higher adjuvant dose (group 1 vs. 4, 20% to 83%; Figure 5, M and N). Memory Env-specific CD8^+^ T cells were only sporadically detectable at week 24 (Figure 5O). Overall, QS-21 SMNP induced robust and durable Env-specific CD4^+^ T cells and some CD8^+^ T cells, and both CD4^+^ and CD8^+^ T cell responses increased with higher QS-21 SMNP doses.

### SMNP dose determined BM plasma cells and neutralizing antibodies.

**Figure 6:**
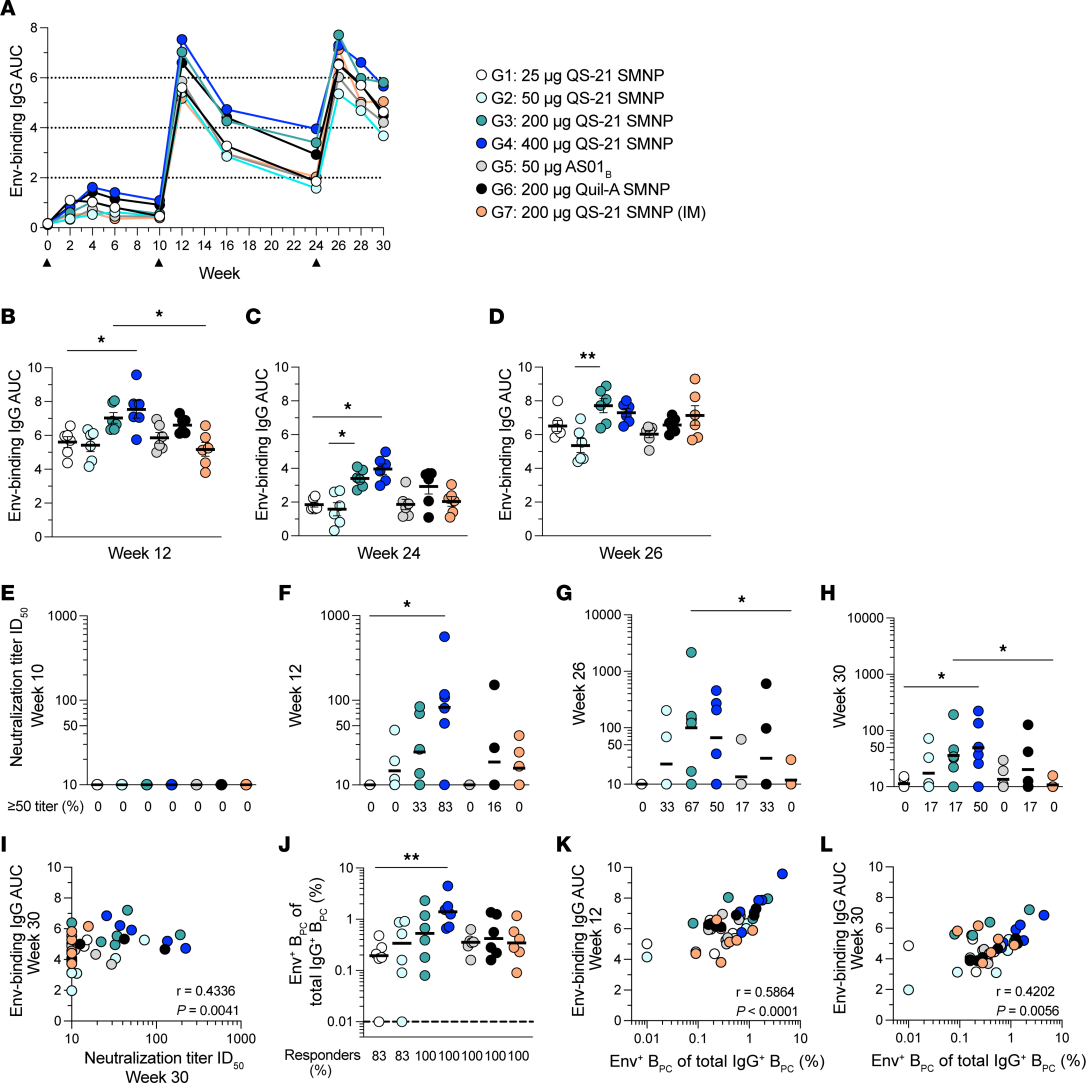
Higher induction of Env-specific BM B_PC_ and neutralizing antibodies with high QS-21 SMNP doses. (**A**) Mean AUC of Env-binding IgG antibodies at different time points after immunization. Black triangles represent the time of immunization. (**B**–**D**) AUC of Env-binding IgG antibodies at week 12 (**B**), week 24 (**C**), and week 26 (**D**). (**E**–**H**) Serum dilution at 50% inhibition of BG505 pseudovirus in a neutralization assay (ID_50_) at week 10 (**E**), week 12 (**F**), week 26 (**G**), and week 30 (**H**). (**I**) Correlation between AUC of Env-binding IgG antibodies and neutralization titers at week 30. (**J**) Frequency of Env^+^ IgG^+^ B_PC_ in BM at week 37. (**K** and **L**) Correlation between AUC of Env-binding IgG antibodies at week 12 (**K**) or week 30 (**L**) and frequency of Env^+^ IgG^+^ B_PC_. Error bars in **B**–**D** represent mean with SEM. Horizontal black lines in **E**–**H** and **J** indicate geometric mean and median, respectively. The dotted black line in **J** indicates the LOD and was used to calculate percent responders. Statistical significance (**B**–**D** and **J**) was assessed using the Kruskal-Wallis test, followed by Dunn’s multiple-comparison test; and in **E**–**H** was assessed using an unpaired 2-tailed Mann-Whitney *U* test. Data in **I**, **K**, and **L** were analyzed using Spearman’s correlation test. *P ≤ 0.05 and **P < 0.01. All data represent *n* = 6 animals/group.

Antibodies are critical components of most vaccine immune responses. Circulating Env-binding IgG responses were longitudinally analyzed for all study groups. Env-binding IgG was detectable after priming (weeks 2–10) in all groups (Figure 6A and Supplemental Figure 6A). Env-binding IgG substantially increased up to 13-fold after boosting (week 12 vs. 10). The 200 and 400 μg QS-21 SMNP doses induced the highest amount of Env-binding IgG after the first boost (week 12 group 1 vs. 4, *P* = 0.036) and after the second boost (week 26, group 2 vs. 3, *P* = 0.0054; Figure 6, B–D, and Supplemental Figure 6, D–F). Importantly, Env-binding IgG decayed across the groups after boosting; however, antibody titer stability (weeks 16–24) in the 400 μg QS-21 SMNP group was slightly better than in the 25 μg QS-21 SMNP group (group 1 vs. 4, *P* = 0.041; Supplemental Figure 6, G and H).

Tier 2 autologous neutralizing responses were assayed after priming (week 10) and after boosts (weeks 12, 26, and 30). No neutralizing responses were detectable in any of the groups after priming, as expected (Figure 6E). After the first boost (week 12), the highest titers were detected in the 400 μg QS-21 SMNP group (group 1 vs. 4, *P* = 0.015; Figure 6F). The 400 μg QS-21 SMNP group also had the highest amount of responding animals (~83%, 5 of 6), followed by the 200 μg QS-21 SMNP group (~67%, 4 of 6) and the 50 μg QS-21 SMNP group (50%, 3 of 6). No neutralizing antibodies were detectable in the 25 μg QS-21 SMNP group or in the 50 μg AS01_B_ group. Compared with s.c. immunization, i.m. immunization was less effective (week 26, group 3 vs. 7, *P* = 0.028; Figure 6G). The overall trends continued to hold at week 30, with a modest decline compared with week 26 (Figure 6H). Env-binding IgG titers correlated significantly with neutralization titers at week 30 (*P* = 0.0041; Figure 6I). Thus, stronger polyclonal neutralizing serum antibody responses were mounted when QS-21 SMNP was delivered s.c. compared with i.m.

BM aspirates were subsequently collected to measure BM B_PC_ from all groups. Env-specific B_PC_ were detected in the majority of animals (Figure 6J). Interestingly, the dose-dependent trends for QS-21 SMNP were consistent with other immune responses, with the 400 μg group inducing the highest amount of Env-specific B_PC_ (group 1 vs. 4, *P* = 0.0045; Figure 6J). BM B_PC_ frequencies correlated significantly with circulating Env-binding IgG titers (weeks 12 and 30, *P* < 0.01; Figure 6, K and L). Overall, 400 μg QS-21 SMNP induced the highest amount of Env-binding IgG, neutralizing antibodies, and BM B_PC_ responses.

## Discussion

This study shows that QS-21 SMNP was a potent adjuvant for recombinant protein immunizations, with a promising safety and reactogenicity profile in NHPs. The SMNP dose regulated the magnitude and quality of the immune memory responses. The lowest tested dose of QS-21 SMNP (25 μg) in this study was capable of inducing immune memory. Doses of up to 400 μg QS-21 SMNP were well tolerated and induced the strongest responses, including neutralizing antibodies and antigen-specific B_Mem_, B_PC_, and T_Mem_. AS01_B_ is an important clinically relevant comparator for QS-21 SMNP. QS-21 SMNP and AS01_B_ elicited comparable immune responses at 50 μg. This is notable given that SMNP contains 10-fold less MPLA than AS01_B_, and it is plausible that SMNP can be coformulated with protein, as is the case with ISCOM-related adjuvants in clinical use (39), whereas AS01_B_ cannot be stored premixed with protein. Despite the lower amounts of MPLA used in SMNP plasma, cytokine responses were comparable to those induced by AS01_B_. It is possible that the nanoparticle structure of SMNP enhances its capacity to induce strong inflammatory responses even with lower MPLA content. Plasma cytokines serve as a benchmark for assessing the adjuvanticity of both adjuvant dose and type, but predicting human reactogenicity based solely on plasma cytokine responses in NHPs remains challenging. We speculate that in humans, reactogenicity to QS-21 SMNP and AS01_B_ may be comparable, potentially limiting the use of certain high-dose administrations despite enhanced immune responses. Overall, QS-21 SMNP is a powerful and potent adjuvant for immune responses associated with protective immunity.

Durability of immune responses is critical to ensure ongoing vaccine protection in the absence of repeated boosters. The data reported herein highlight that SMNP has the ability to induce durable B and T cell memory. Several lines of evidence indicate better immune memory with higher QS-21 SMNP doses. Env-specific CD4^+^ T_Mem_ and B_PC_ expression was more robust and reliable in the 400 μg QS-21 SMNP animals compared with the 25 μg QS-21 SMNP group. Furthermore, Env-binding IgG responses had less decay after boosting (weeks 16–24) in the 400 μg QS-21 SMNP group, indicating more-durable circulating antibody responses. The strong correlation between Env-binding B_PC_ and Env-binding IgG further supported these findings. Env-binding B_Mem_ frequencies decayed with similar kinetics in high- and low-dose QS-21 SMNP groups after boosting, but the 400 μg QS-21 SMNP group had higher frequencies compared with the 25 μg QS-21 SMNP at week 24. These results indicate that SMNP induces long-lasting dose-dependent Env-binding B_Mem_ responses after immunization.

While the magnitude of Env-binding B_Mem_ was higher with 400 μg QS-21 SMNP compared with 50 μg QS-21 SMNP after the booster immunization, single B cell sequencing revealed similar qualities early after the boost in both cases. Based on the similarities of Env-binding B_Mem_ induced with different doses of QS-21 SMNP, it is plausible that their affinities were generally comparable as well. Antigen-specific B_Mem_ can increase their SHM after 2 immunizations in NHPs and humans (40–42). This phenomenon may predominantly occur through the recruitment of B_Mem_ into GCs during recall responses (18, 43). The 50 and 400 μg QS-21 SMNP groups may have had different GC longevities, which could have affected the level of recall responses and thereby the diversity and quality of Env-binding B_Mem_ at later time points. The induction of more abundant B_Mem_ clonotypes in the 400 μg QS-21 SMNP group compared with the 50 μg group may also have increased the chance of recruiting rare clones with neutralizing potential into the response. In general, vaccine strategies capable of inducing durable immune responses will be facilitated by in-depth understanding of how different antigen-specific B and T cell compartments are induced and maintained.

Protective vaccine responses generally require the induction of robust antigen-specific T cell activity. AS01 adjuvants, including AS01_B_, were selected for advancement to clinic based on their ability to induce enhanced cellular immunity in addition to humoral immunity (26). T cell response induction by SMNP was of particular interest for this study. We found Env-specific CD4^+^ T cells were primed at all SMNP doses. Consistently high CD4^+^ T cell responses were elicited with SMNP doses of at least 50 μg. Env-specific memory CD4^+^ T cells were generated at all SMNP doses. Env-specific memory cT_FH_ and CD4^+^ T cells producing IFN-γ or IL-21 were more abundant with higher QS-21 SMNP doses. Overall, SMNP at the doses tested was capable of inducing substantial frequencies of antigen-specific CD4^+^ T cells, with robust functionalities, indicative of a substantial capacity to aid with vaccine antibody responses and potentially protective immunity.

Env-specific CD8^+^ T cells were also detected in this study. Env-specific CD8^+^ T cells varied among the study groups, but were primed more consistently with s.c. delivery of QS-21 or Quil-A SMNP doses of at least 200 μg. More work is required to determine how protein immunogen with adjuvants prime antigen-specific CD8^+^ T cells and what specific role these cells may play in protective immunity.

We administered immunizations unilaterally in this study to model the most common situation in human vaccination. The neutralizing responses after unilateral administrations here for the highest-SMNP-dose group (400 μg QS-21 SMNP) were slightly lower than the previously published responses to bilateral administration of 375 μg (187.5 μg/side) or 750 μg (375 μg/side) Quil-A SMNP with equivalent doses of BG505 MD39 (8, 22). Theoretically, bilateral immunizations can reach twice as much LN engagement than unilateral immunizations, thus increasing the odds of recruiting rare clones with neutralizing capabilities. Although the dose and saponin source are not identical in these studies, the outcomes indicate that it is plausible that bilateral immunization increases neutralizing antibody responses. However, this needs to be directly tested in future studies. Subcutaneous administrations showed stronger responses than i.m. administrations, including neutralizing antibody responses. Subcutaneous drainage is less restricted than i.m. drainage (44, 45), which can increase LN engagement. Although the anatomical site (thigh vs. arm) differs here, this finding is consistent with previous reports showing lower tier 2 autologous neutralizing response via i.m. than s.c. delivery in the thighs of NHPs (44). In sum, s.c. delivery of QS-21 SMNP compared with i.m. delivery led to stronger acute CD4^+^ T cell responses and neutralizing antibody responses.

Recent advances in QS-21 production using tobacco plants or yeast as a heterologous expression system instead of the Chilean soapbark tree to provide sustainable sources for this rare and crucial molecule are encouraging (46, 47). Although we show that QS-21 was an excellent saponin in terms of potency and safety to formulate in SMNP, the proven safety of Quil-A in formulations with phospholipids and cholesterol in animal models should not be neglected. This potentially suggests other, more abundant saponins could be considered for their applications in vaccine adjuvants. It would be worthwhile to compare different saponin fractions in SMNP formulation to investigate the potential different roles of QS-21 and other fractions on immune responses and their linked mechanism. Initial innate immune responses are likely important in the optimal induction of these durable adaptive immune responses. A better understanding of the mechanism of action of adjuvants will be important for building an arsenal of different immune activators to generate durable responses.

There were certain limitations in this study. We investigated different adjuvant doses, but not different immunogen doses. The HIV immunogen tested here is a model for difficult antigens with dense glycan shields. Long-term durability of immune responses more than 1 year after vaccination was not studied here and needs to be investigated in future studies.

In sum, our results are promising and encouraging for the development of QS-21 SMNP as a viable and safe vaccine adjuvant. Human use of QS-21 SMNP is currently being investigated with a germline-targeting HIV vaccine, N332-GT5 (ClinicalTrials.gov NCT06033209).

## Methods

### Sex as a biological variable.

Our study examined male and female animals, and similar findings are reported for both sexes.

### Adjuvants.

Saponins Quil-A (vac-quil) and QS-21 were purchased from InvivoGen and Desert King, respectively, and used as received. Phosphorylated hexaacyl disaccharide (PHAD) MPLA was obtained from Avanti. Laboratory-scale SMNPs were prepared as previously described (8). AS01_B_ was obtained from the Shingrix (GlaxoSmithKline) adjuvant vials that are provided separately from the antigen vials for the vaccine. GMP-process QS-21 SMNP was prepared using a tangential flow filtration–based (TFF-based) detergent dilution process (27). Briefly, SMNP components were solubilized in 20% (wt/vol) MEGA-10 surfactant at a 10:2:1:1 mass ratio of QS-21/cholesterol/MPLA/DPPC at a batch size of 500 mg QS-21. This starting detergent/lipid solution was diluted with PBS (pH 7.4) to reach a final QS-21 concentration of 5 mg/mL and MEGA-10 concentration of 7.5%. The adjuvant/lipid mixture was then diluted stepwise with PBS (pH 7.4), with sufficient incubation time between each dilution step to allow for controlled SMNP self-assembly (Supplemental Table 2). After SMNP assembly, the sample was concentrated 20-fold on a 100 kDa hollow fiber TFF membrane (mPES, surface area 0.16 m^2^, Repligen). After concentration, the sample underwent continuous diafiltration for 10 diafiltration volume equivalents against PBS pH 6.5. The sample was then filtered through a 0.2 μm membrane and used to fill 3 mL 2R Type I USP borosilicate glass vials with 0.6 mL sample. Vials were closed with 13 mm bromobutyl 4023/50, FluroTec-coated, B2-40 gray stoppers and a 13 mm aluminum caps with green plastic flip-off. Nanoparticle micrographs were acquired using transmission electron microscopy (TEM) on a JEOL 2100F microscope (200 kV) with a magnification range of 10,000×–60,000×. All images were recorded on a Gatan 2kx2k UltraScan CCD camera. Negative-stain sample preparation was performed by allowing 10 μL of SMNP to adsorb on a 200-mesh copper grid coated for 1 minute. The excess solution was then removed by touching the grid with a Kimwipe. The negative staining solution, 10 μL phosphotungstic acid (PTA) 1% aqueous, was then quickly added, and the excess solution was removed with a Kimwipe. After drying at room temperature, the grid was mounted on a JEOL single-tilt holder equipped in the TEM column for image capture.

### Protein production.

BG505 MD39 untagged trimer immunogens and His-Avi–tagged biotinylated trimers (BG505 MD39 and BG505 MD39-bKO) (18) were produced by transient cotransfection of HEK293F cells (Thermo Fisher Scientific R79007) with furin as previously described (28). His-Avi–tagged trimers were purified by immobilized metal ion affinity chromatography (IMAC) using HisTrap excel columns (Cytiva), followed by size-exclusion chromatography (SEC) using a Superdex 200 Increase 10/300 GL column (Cytiva). His-Avi–tagged trimers were biotinylated using BirA (Avidity) according to the manufacturer’s instructions and purified again to remove excess biotin using SEC. BG505 MD39 untagged trimer immunogens were purified by 2G12 affinity chromatography, followed by SEC. Immunogen preparations were confirmed to contain less than 5 EU/mg endotoxin using an Endosafe instrument (Charles River).

### Animals, immunizations, and sample collections.

Indian rhesus macaques (*Macaca mulatta*) were bred and housed at the UC Davis California National Primate Research Center and maintained in accordance with NIH guidelines. Animal age, body weight, and sex were equally distributed among groups. Animals ranged in age from 3 to 13 years (median of 5 years), and each group contained 3 females and 3 males (*n* = 6 per group). Immunizations were prepared in sterile PBS with 100 μg BG505 MD39 HIV envelope immunogen and the appropriate amount of adjuvant. Adjuvants administered differed in formulation, dose, and delivery routes among groups. The following adjuvants with coadministration of 100 μg BG505 MD39 HIV immunogen were given: Group 1 received 25 μg QS-21 SMNP; group 2 received 50 μg QS-21 SMNP; group 3 received 200 μg QS-21 SMNP; group 4 received 400 μg QS-21 SMNP; group 5 received 50 μg AS01_B_; group 6 received 200 μg Quil-A SMNP; and group 7 received 200 μg QS-21 SMNP. The adjuvant dose relates to the quantity of saponin administered. The total amount of QS-21 was consistent between the 50 μg AS01_B_ and QS-21 SMNP formulations. Injections in groups 1–6 were given s.c. into the inner thigh (1 mL per injection) and in group 7 was given i.m. into the deltoid (0.5 mL per injection). Animals were immunized a total of 3 times at weeks 0, 10, and 24 as a unilateral bolus injection on the left side. All experimental manipulations (immunizations and sample collections) were performed under ketamine anesthesia (10 mg/kg body weight; i.m.). Blood samples were collected via peripheral venipuncture. Plasma and serum samples, PBMCs, and LN mononuclear cells (from FNAs) were processed and cryopreserved according to standard laboratory protocols and subsequently shipped for downstream assays. BM aspirates were collected with heparinized syringes, placed in conical tubes containing R10 media, and shipped overnight at 4°C for downstream assays.

### Detection of antigen-binding or total B_Mem_, B_GC_, and GC-T_FH_ by flow cytometry.

LN FNAs or PBMCs were thawed in RPMI media (Corning, 10-041-CV) containing 10% FBS (GeminiBio, 900-108), 1× penicillin/streptomycin (Thermo Fisher Scientific, 10378-016), and 1× GlutaMAX (Thermo Fisher Scientific, 35050-06). Biotinylated BG505 MD39 or BG505 MD39-bKO was tetramerized by streptavidin conjugated to a fluorescent label (SA). Biotinylated BG505 MD39 was tetramerized with SA-BV421 or SA-AF647. Biotinylated BG505 MD39-bKO was tetramerized with SA-PE. All SA conjugates were added in a stepwise addition by adding one-third of the SA to the biotinylated protein at a time and incubating for 15 minutes in between. PBMCs or LN FNAs were plated in a 96-well U-bottom plate at up to 5 × 10^6^ cells per well, stained with 1:25 Fc block (BD Biosciences 564220), and washed with FACS buffer (2% FBS in PBS). Cells were first incubated with BG505 MD39-bKO probe for 20 minutes and then with BG505 MD39 probes for 30 minutes at 4°C in the dark. Cells were then stained with surface antibodies shown in Supplemental Tables 3–5 depending on the sample type and incubated for an additional 30 minutes at 4°C in the dark. Following incubation, cells were washed twice with FACS buffer and acquired on a 5-laser-equipped Aurora (Cytek Biosciences) spectral flow cytometer. Anti-CD38–PE-Cy5 was conjugated using purified anti-CD38 (OKT10; Nonhuman Primate Reagent Resource [NHPRR] PR-0056) and the PE/Cy5 Conjugation Kit (Abcam ab102893). Anti-CD71–PE-CF594 was a custom order from BD Biosciences, with anti-CD71 (L01.1; 347510) conjugated to PE-CF594.

For analysis of bulk B_GC_ and GC-T_FH_, thresholds of 250 B cells and 500 CD4^+^ T cells, respectively, were used. For analysis of Env-binding B_GC_, a threshold of 75 B_GC_ cells was used. For Env-binding B_Mem_ in LN FNAs, samples with at least 1,000 B cells were included for analysis. For analysis of bKO^+^ Env-binding B_Mem_ in the blood, a threshold of 10 Env^+^ B_Mem_ was used. The limit of detection (LOD) for Env-binding B_GC_ and B_Mem_ in LN FNAs was 0.01% and was determined by calculating the median of (3/[number of B cells recorded]) from preimmunized samples. The LOD for Env-binding B_Mem_ in the blood was 0.0006% and was determined by calculating the median plus 2× SD of (1/[number of B cells recorded]) from preimmunized samples. Any sample that met the above threshold criteria and fell below 0.001% was set to a baseline of 0.001%. To calculate B_Mem_ half-life (*t*_1/2_) between weeks 12 and 16 or weeks 16 and 24, we log_2_ transformed data and utilized simple linear regression.

### Single B cell sequencing.

Env-binding B_Mem_ at week 12 from the 50 μg and 400 μg QS-21 SMNP groups were stained with tetramerized BG505 MD39 probes using fluorescent-SA and TotalSeq-C oligonucleotide-tagged fluorescent-SA surface antibodies and 6 different TotalSeq-C oligonuceleotide-tagged anti-β2M and -CD298 antibodies (BioLegend 394661, 394665, 394673, 394675, 394677, and 394679) to multiplex animal samples. TotalSeq-C BV421 SA was a custom order from BioLegend, with SA conjugated to both the BV421 fluorophore and the oligonucleotide. The antibody panel and reagents are summarized in Supplemental Table 6. Samples were sorted on a FACSymphony S6 (BD Biosciences) into 1.5 mL tubes with FBS supplemented RPMI media. After sorting, samples were washed once with PBS and loaded onto a Chromium chip and controller according to the manufacturer’s recommendation (10x Genomics). VDJ, GEX, and HTO libraries were generated and sequenced on a NovaSeq 6000 (Illumina). Cell Ranger v7.2.0 (10x Genomics) was used to de-multiplex sequencing files into FASTQ files and assemble reads from the libraries. VDJ reads were assembled de novo in Cell Ranger, and contigs were aligned to a custom *Macaca mulatta* germline VDJ reference using IgBLAST and the Change-O 1.3.0 package from the Immcantation framework (48). Sequences were assigned to animals using the MULTIseqDemux function in Seurat v5 (49). Inferred germline sequences (CreateGermline.py) and clones (DefineClones.py) were determined with Change-O. SHMs were quantified using the observedMutations command in SHazaM package v1.2.0 comparing heavy-chain (HC) or light-chain (LC) sequences to the inferred germline sequences (masking the D gene sequences). To calculate amino acid mutation frequency, HC or LC sequences from germline_alignment_d_mask and sequence_alignment were translated into protein sequences and compared. Only sequences matching the germline in length were included in the analysis, with mutations defined as amino acid differences at corresponding positions. To determine clonal abundance, clonotypes for each animal were quantified using the countClones function in Alakazam package v1.3.0. Clonal richness (Chao1) was calculated using the iNEXT package v3.0.0 in R (50). Gene expression integrative analysis was done with Seurat v5 using CCAIntegration. Cells expressing fewer than 200 or more than 4,500 transcripts and more than 5% mitochondrial genes were excluded. Clusters containing non-B cells were removed for analysis.

### Detection of antigen-specific CD4^+^ or CD8^+^ T cells by flow cytometry.

AIM and ICS assays for the detection of antigen-specific T cells was performed as previously described (22). Cryopreserved PBMCs were thawed and washed in RPMI media containing 10% FBS, 1× penicillin/streptomycin, and 1× GlutaMAX (R10 media). Cells were counted and plated at 1 × 10^6^ cells per well in a round-bottom 96-well plate. Prior to stimulation, cells were blocked with 0.5 μg/mL anti-CD40 mAb (Miltenyi Biotec 130-094-133) and stained with anti-CXCR5 and -CCR7 for 15 minutes at 37ºC. Then, cells were incubated for 24 hours in the presence of: (a) 5 μg/mL BG505 MD39 Env peptide pool; (b) 1 ng/mL staphylococcal enterotoxin B (SEB) used as a positive control; or (c) an equimolar amount of DMSO as negative, unstimulated control plated in duplicate. BG505 MD39 Env peptide pools consisted of overlapping 15-mer peptides that covered the entire protein sequence and were resuspended in DMSO. After 24 hours of incubation, intracellular transport inhibitors – 0.25 μL/well GolgiPlug (BD Biosciences 555029) and 0.25 μL/well GolgiStop (BD Biosciences 554724) – and AIM marker antibodies (CD25, CD40L, CD69, OX40, 4-1BB) were added to the samples and incubated for another 4 hours. Cells were then centrifuged and incubated for 15 minutes at room temperature (RT) with Fc Block (BioLegend 422302) and Fixable Live/Dead Blue (Thermo Fisher Scientific). Cells were then washed with FACS buffer (2% FBS in PBS) and stained with the surface antibodies for 30 minutes at 4°C. Next, cells were fixed with 4% formaldehyde, permeabilized with a saponin-based buffer, and stained with intracellular cytokine antibodies for 30 minutes at RT. Cells were washed with PBS and stored at 4°C until being acquired by a 5-laser-equipped Aurora spectral flow cytometer. The antibody panel and reagents are summarized in Supplemental Table 7. For data analysis, antigen-specific T cells were measured as background-subtracted data, where the linear averages of the DMSO background signal, calculated from duplicate wells for each sample, were deducted from the stimulated signal. A minimum threshold for DMSO background signals was set at 0.005%, and the limit of quantitation (LOQ) was defined as the geometric mean of all DMSO-negative control wells. For each sample, the stimulation index (SI) was calculated as the ratio between the AIM^+^ response in the stimulated condition and the average DMSO response for the same sample. Samples with an SI lower than 2 for CD4^+^ or 3 for CD8^+^ T cells responses and/or with a background-subtracted response lower than the LOQ were considered as nonresponders. Nonresponder samples were set to 2-fold below the LOQ. Sample 48005 from group 1 at week 2 was excluded from antigen-specific T cell analysis due to the viability of the cells.

### ELISA.

Plasma samples from animals were serially diluted (starting at 1:50) and plated on pre-coated wells with BG505 MD39 antigen. Fort this, 96-well plates (Corning 3690) were coated with SA (Thermo Fisher Scientific 434302) first, blocked with 2% BSA and then incubated with biotinylated BG505 MD39. Env binding-IgG or -IgA antibodies were detected using HRP goat anti-human IgG (Jackson ImmunoResearch 109-035-098) or HRP goat anti-rhesus IgA (NHPRR 00365) and 1-Step Ultra TMB ELISA Substrate Solution (Thermo Fisher Scientific 34029). The reaction was stopped with 2N of sulfuric acid (Ricca Chemical 8310-32) and read at 450 nm on a FlexStation 3 plate reader (Molecular Devices).

### Neutralization assays.

Neutralization assays using the BG505.W6M.ENV.C2 isolate (T332N) pseudovirus were performed as previously described (44). Assays were run in duplicate by researchers masked to the experimental protocol. Initial serum dilution was set at 1:10, and values below this were considered as nonneutralizing. Absolute ID_50_ values were calculated using normalized relative luminescence units and a customized nonlinear regression model with the bottom constraint set to 0 and top constraint set to <100:
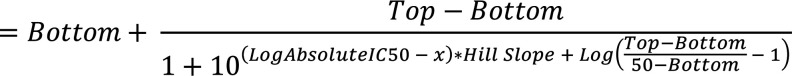


### ELISPOT.

BM aspirates were processed to harvest mononuclear cells, which were serially diluted and plated onto precoated wells with BG505 MD39 antigen (bound via Galanthus Nivalis Lectin [Vector Laboratories L-1240]) or total goat anti-human Ig antibody (Southern Biotech 2010-01) as captures. Plates were incubated in a 5% CO_2_ incubator at 37°C for 16 hours. Secreted antibodies were detected using biotinylated goat anti-human IgG (Southern Biotech 2045-08), HRP Avidin D (Vector Laboratories A-2004), and AEC substrate (Acros Organics 147870250). Formed spots were imaged using the ImmunoSpot CTL counter and Image Acquisition 4.5 software (Cellular Technology), and counted manually.

### Statistics.

Statistical analyses were performed using GraphPad Prism 10, with specific tests indicated in the figure legends. Prior to the start of the study, we hypothesized that a greater neutralization response would be induced in the highest-dose QS-21 SMNP group (group 4, 400 μg) than in the lowest-dose QS-21 SMNP group (group 1, 25 μg) and that s.c. QS-21 SMNP administration (group 3, 200 μg) would elicit a greater neutralization response than i.m. administration (group 7, 200 μg). For statistical analyses of the neutralization data based on these specific, prespecified hypotheses and comparisons involving only 2 groups, we used an unpaired 2-tailed Mann-Whitney *U* test. For most other analyses involving multiple-group comparisons or exploratory assessments, statistical tests were subject to multiple-comparison corrections. *P* ≤ 0.05 was considered significant, with the following significance levels: NS *P* > 0.05, *P ≤ 0.05, **P < 0.01, ***P < 0.001, ****P < 0.0001.

### Study approval.

All animal husbandry and sample collection procedures were in compliance with the *Guide for the Care and Use of Laboratory Animals* (National Academies Press, 2011); and were performed according to the standard operating procedures of and with approval by the Institutional Animal Care and Use Committee of the University of California, Davis.

### Data availability.

All data underlying the conclusions of this work are presented in the main figures, supplemental material, and Supporting Data Values file. The scRNA-Seq data sets were deposited in the NCBI’s Gene Expression Omnibus database (GEO GSE291681).

## Author contributions

DJI and SC designed the original animal study. KKAVR and PRR adapted and supervised the animal study. DJI, SC, and PRR designed the majority of the experiments. PRR, AM, and NIB performed and analyzed B cell responses in the blood and LNs. EMZ designed, performed, and analyzed T cell assays with technical assistance from PGL. LM and KKM performed and analyzed plasma cytokine and antibody responses assays. ISP performed and analyzed TEM. MBM and AAL performed and analyzed antibody response experiments. IB, LH, and DRB performed and analyzed serum neutralization assays. AP and SPK performed and analyzed BM plasma cell response experiments. WRS, CAC, and BG supplied immunogens and reagents. SP, ES, and SLS supervised GMP-process SMNP production. AW, GS, MB, and DK supplied GMP-process SMNP. PRR wrote the original draft with substantial input from SC, DJI, and AM. WRS, DRB, EMZ, KKAVR, SPK, AP, and CAC edited the manuscript. All authors read and approved the manuscript.

## Supplementary Material

Supplemental data

Supporting data values

## Version 1 03/04/2025

In-Press Preview

## Version 2 04/15/2025

Electronic publication
