# Supplementary material for: The saponin monophosphoryl lipid A nanoparticle adjuvant induces dose-dependent HIV vaccine responses in nonhuman primates
Source: J Clin Invest. 2025 Mar 4;135(8):e185292. doi: 10.1172/JCI185292 (PMC11996878; doi:10.1172/JCI185292)
Supplement: Supplemental data [file jci-135-185292-s092.pdf]

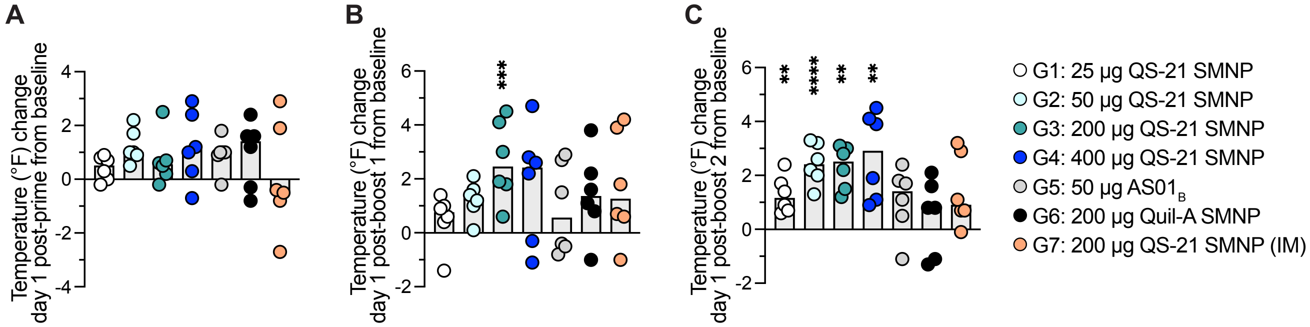

Supplemental Figure 1. Animal body temperature changes post-immunizations. (**A-C**) Change in rectal body temperature (°F) from baseline to day 1 (**A**) post-prime, (**B**) post-boost 1 and (**C**) post-boost 2. Gray bars represent the median. Statistical significance was evaluated using the Friedman test, followed by Dunn's multiple comparisons test, comparing baseline temperatures to post-injection temperatures at day 1 within each group. Significance levels were indicated as **P* ≤ 0.05, ***P* < 0.01, ****P* < 0.001, and *****P* < 0.0001. All data show n = 6 animals/group.

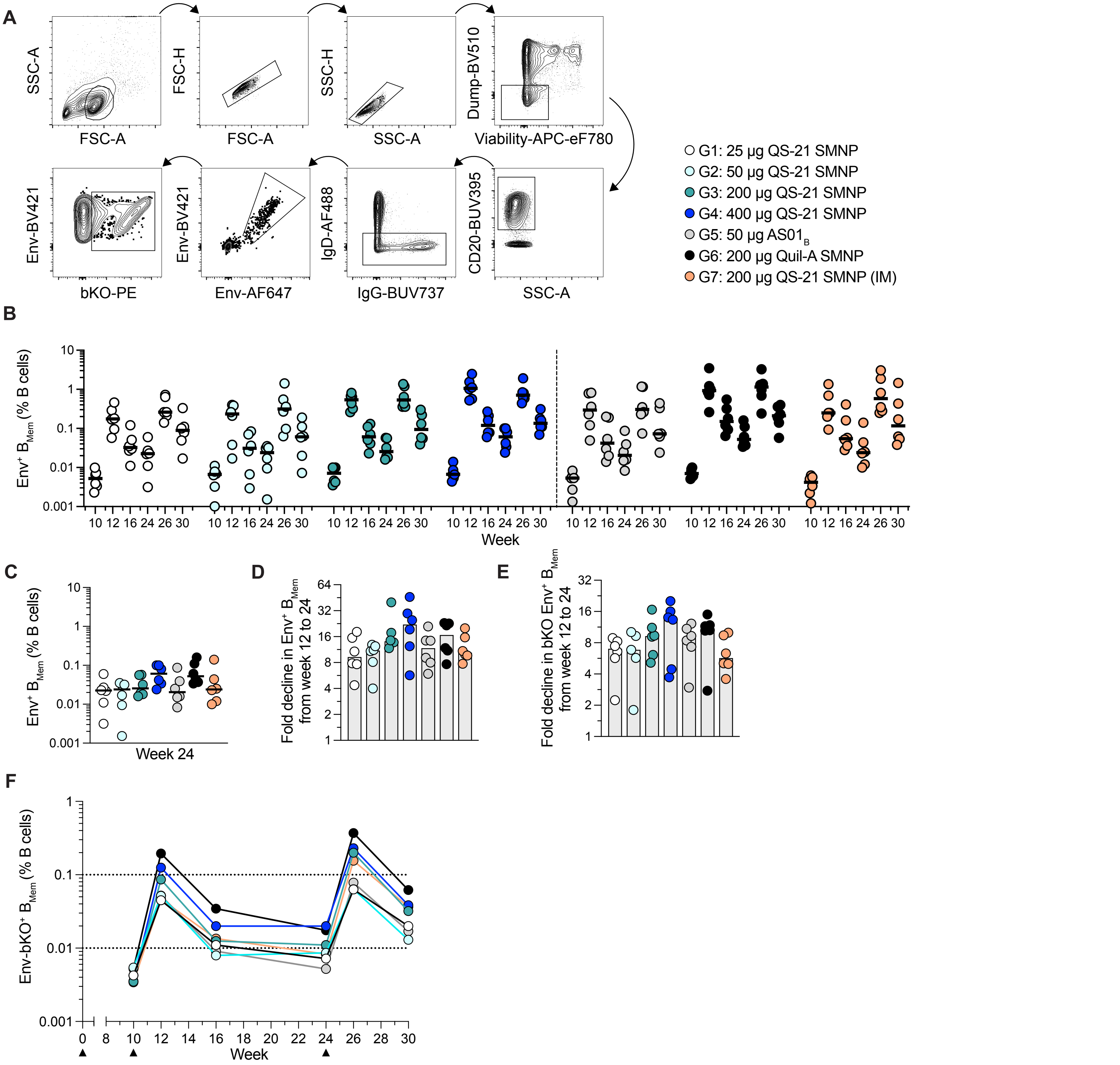

Supplemental Figure 2. Gating strategy of Env-binding B_{Mem} in PBMCs. (**A**) Flow cytometry gating strategy to define Env-binding B_{Mem} in the blood. (**B**) Frequency of Env-binding B_{Mem} over time. (**C**) Frequency of Env-binding B_{Mem} in PBMCs at week 24. (**D**) Fold change in Env-binding B_{Mem} from week 12 to week 24. (**E**) Fold change in bKO-binding B_{Mem} from week 12 to week 24. (**F**) Median frequency of bKO-binding B_{Mem} as a percentage of B cells. Black triangles denote the time of immunization. Horizontal black bars in (**B**) and gray bars in (**C**) and (**E**) represent the median. Statistical significance was assessed using the Kruskal-Wallis test, followed by Dunn's multiple comparisons test. Significance levels were indicated as *$P \leq 0.05$, **$P < 0.01$, ***$P < 0.001$, and ****$P < 0.0001$. All data show n=6 animals/group.

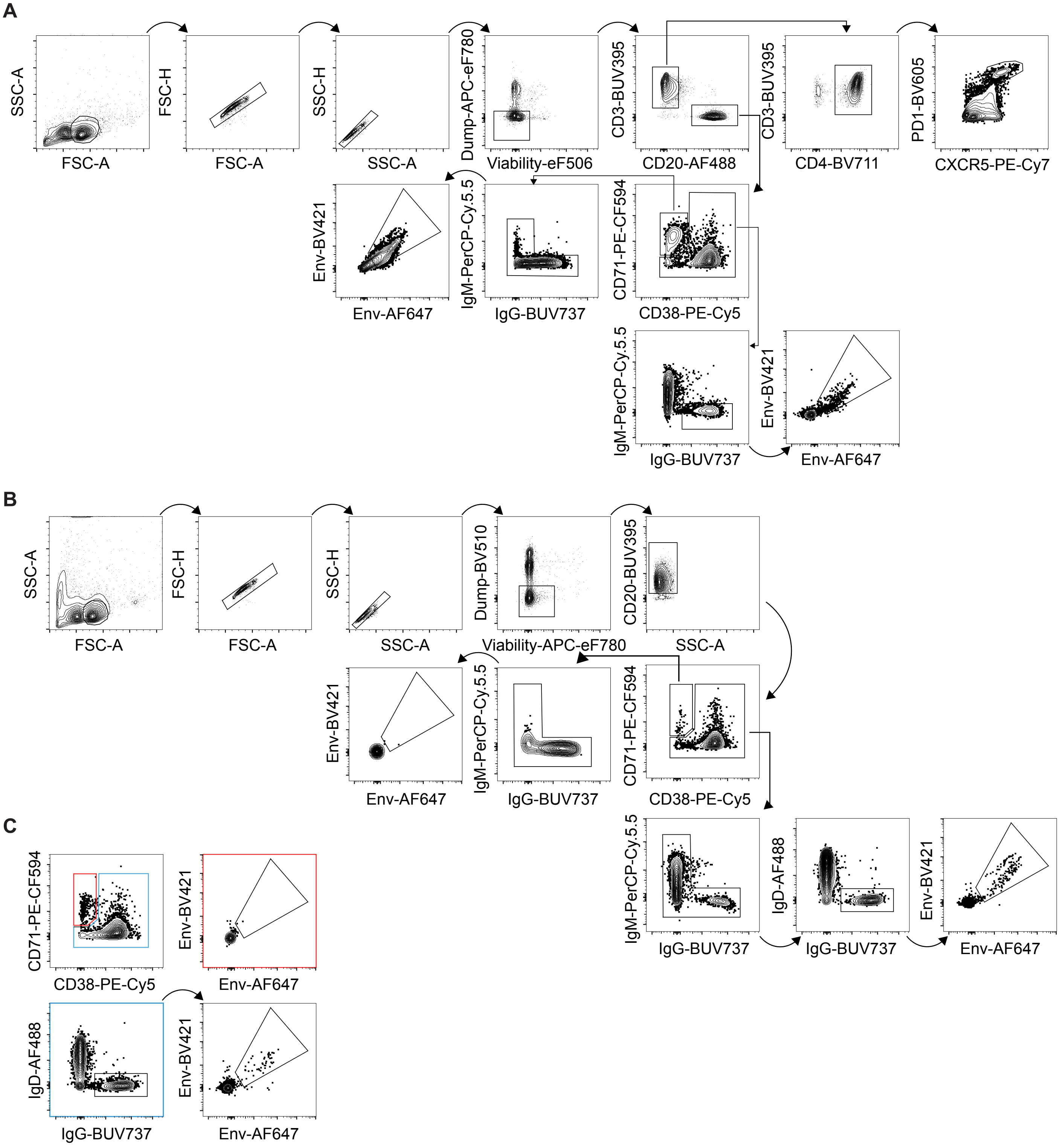

Supplemental Figure 3. Gating strategy of Env-binding B_{GC} and B_{Mem} in LN FNAs. (**A**) Flow cytometry gating strategy to define Env-binding B_{GC} and B_{Mem} in ipsilateral LN FNAs. (**B**) Flow cytometry gating strategy to define Env-binding B_{GC} and B_{Mem} in contralateral LN FNAs. (**C**) Flow cytometry gating of Env-binding B_{GC} ($CD71^+CD38^-$) and B_{Mem} (non-B_{GC} IgD^-IgG^+) cells in contralateral LN FNAs at week 13.

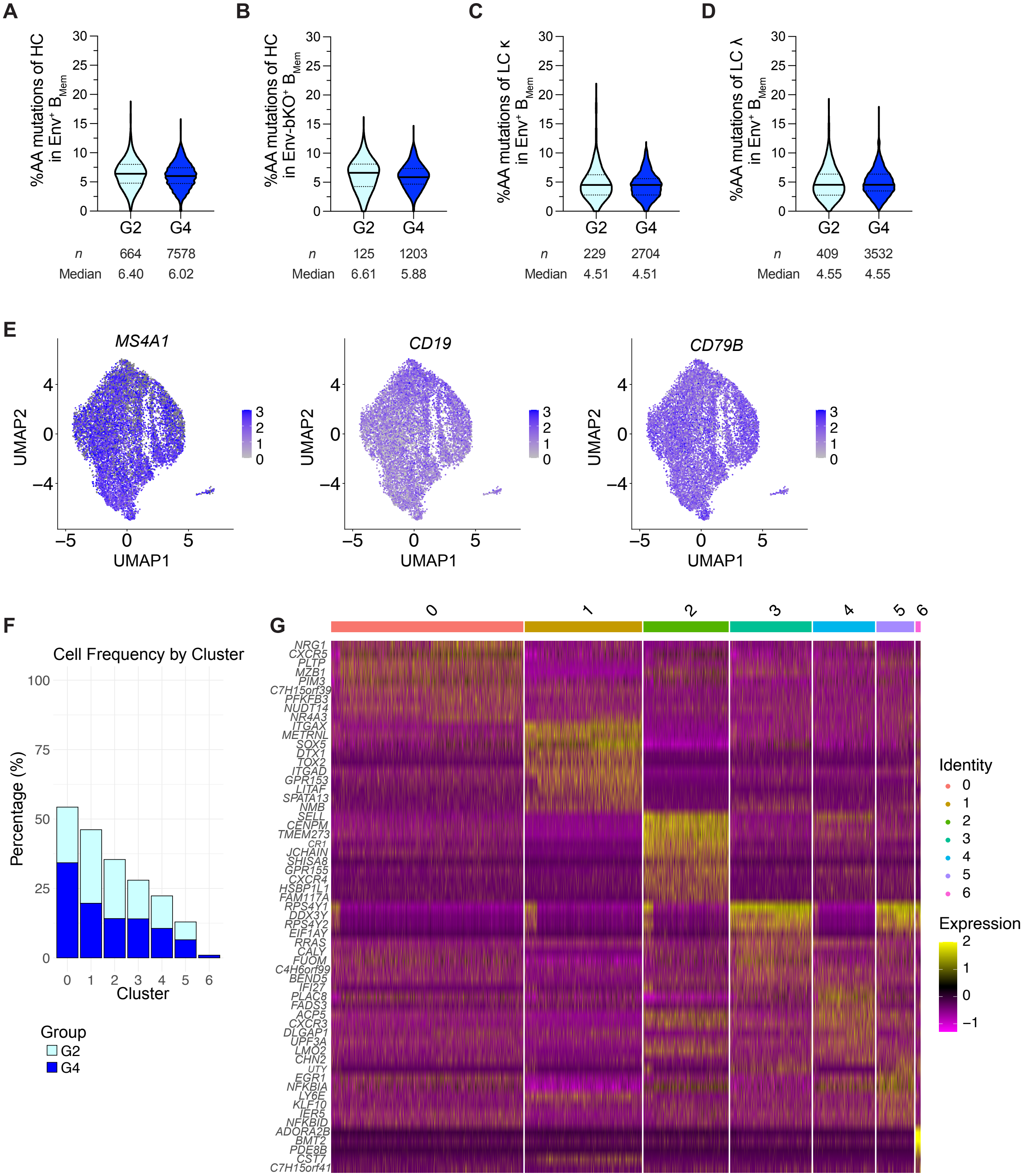

Supplemental Figure 4. Gene expression clusters of Env-binding B_{Mem}. (**A** and **B**) Percent of heavy chain (HC) amino acid (AA) mutations in (**A**) Env-binding B_{Mem} and (**B**) bKO-binding B_{Mem} cells at week 12 in PBMCs. (**C** and **D**) Percent of light chain (LC) (**C**) kappa and LC (**D**) lambda amino acid (AA) mutations in Env-binding B_{Mem} at week 12. (**E**) Uniform manifold approximation and projection (UMAP) of single-cell gene expression profiles for *MS4A1*, *CD19* and *CD79B* in Env-binding B_{Mem} at week 12. (**F**) Cell frequency by cluster in Group 2 (50 µg QS-21 SMNP) and Group 4 (400 µg QS-21 SMNP) of Env-binding B_{Mem}. (**G**) Differential gene expression among clusters of B_{Mem} at week 12. The solid black line and dotted lines in (**A-D**) represent the median and quartiles, respectively. Statistical significance was determined by an unpaired two-tailed Mann-Whitney test. All data show n = 6 animals/group.

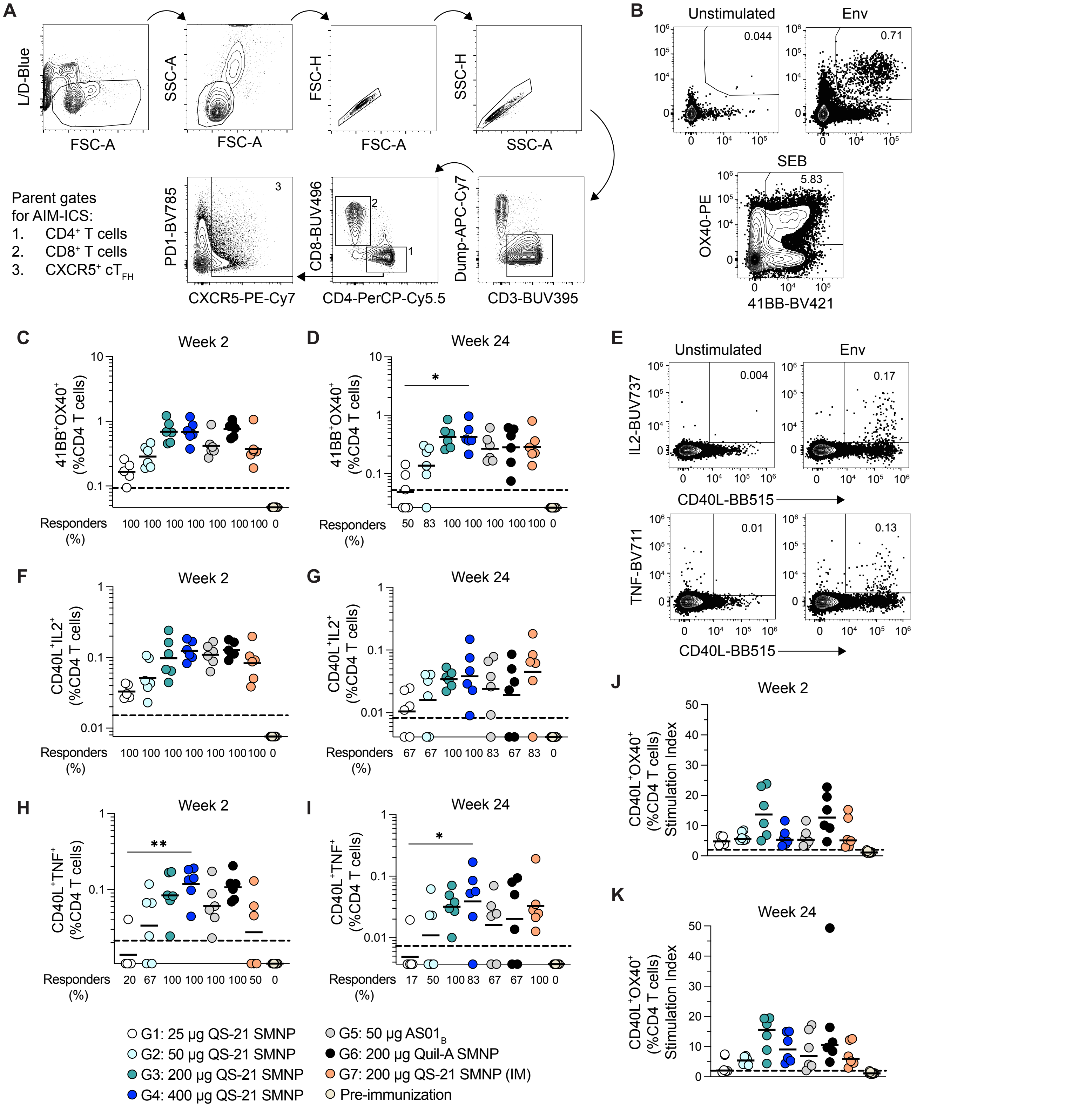

Supplemental Figure 5. Gating strategy of Env-specific CD4 and CD8 T cells. (**A**) Flow cytometry gating strategy to analyze Env-specific CD4 T cells, CD8 T cells and cT_{FH}. (**B**) Representative flow cytometry plots of AIM$^+$ OX40$^+$41BB$^+$ CD4 T cells from unstimulated (negative control), Env peptide pool-stimulated samples or superantigen staphylococcal enterotoxin (SEB, positive control) at week 2. (**C** and **D**) Frequency of AIM$^+$ OX40$^+$41BB$^+$ Env-specific CD4 T cells at (**C**) week 2 and (**D**) week 24. (**E**) Representative flow cytometry plots of CD40L$^+$IL-2$^+$ CD4 T cells and CD40L$^+$TNF$^+$ CD4 T cells from unstimulated or Env peptide pool-stimulated samples at week 2. (**F** and **G**) Frequency of CD40L$^+$IL-2$^+$ Env-specific CD4 T cells at (**F**) week 2 and (**G**) week 24. (**H** and **I**) Frequency of CD40L$^+$TNF$^+$ Env-specific CD4 T cells at (**H**) week 2 and (**I**) week 24. (**J** and **K**) Stimulation index of AIM$^+$ (OX40$^+$CD40L$^+$) CD4 T cells at (**J**) week 2 and (**K**) week 24. Horizonal black lines represent the geometric mean. Black dotted lines indicate the limit of quantification (LOQ). Percent responders was calculated as the percent of animals above the LOQ. Frequencies are shown after subtraction from paired unstimulated samples. Statistical significance was assessed using the Kruskal-Wallis test, followed by Dunn's multiple comparisons test. Significance levels were indicated as $*P \leq 0.05$, $**P < 0.01$, $***P < 0.001$, and $****P < 0.0001$. All data show n = 6 animals/group except Group 1 at week 2 has n = 5 and pre-immunization n = 14 animals.

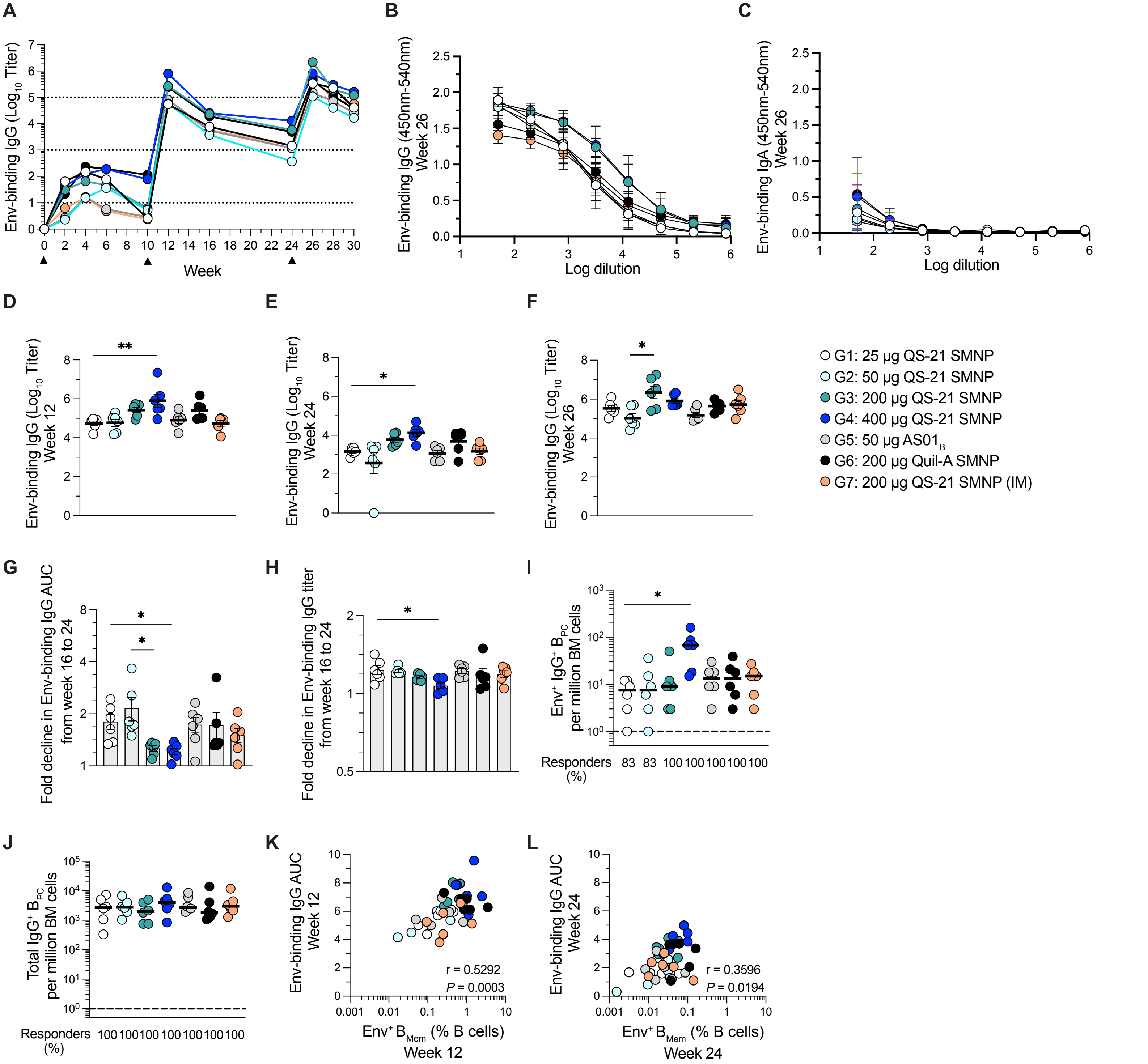

Supplemental Figure 6. Env-binding IgG antibody titer responses. (**A**) Mean Env-binding IgG endpoint titers. (**B-C**) Env-binding antibody curves at week 26 for (**B**) IgG and (**C**) IgA. (**D-F**) Env-binding IgG endpoint titers at (**D**) week 12, (**E**) week 24 and (**F**) week 26. (**G**) Fold change in Env-binding IgG AUC from week 16 to week 24. (**H**) Fold change in Env-binding IgG endpoint titer from week 16 to week 24. (**I**) Env-binding B_{PC} (IgG^+) per million bone marrow cells at week 37. (**J**) Total B_{PC} (IgG^+) per million bone marrow cells at week 37. (**K** and **L**) Correlation between Env-binding IgG antibodies and Env-binding B_{Mem} at (**K**) week 12 and (**L**) week 24. Error bars in (**D-H**) represent mean with SEM. Horizontal black bars in (**I** and **J**) represents the median. Dotted line in (**I** and **J**) denotes the limit of detection (LOD) and was used to calculate percent responders. For (**D-J**) statistical significance was assessed using the Kruskal-Wallis test, followed by Dunn's multiple comparisons test. Data in (**K** and **L**) was analyzed using a Spearman's Correlation test. Significance levels were indicated as $^*P \leq 0.05$, $^{**}P < 0.01$, $^{***}P < 0.001$, and $^{****}P < 0.0001$. All data show n = 6 animals/group.

Supplemental Table 1. GMP-process SMNP characterization

**Characteristic**	**Result**
appearance	Colorless, slightly opalescent
Content QS-21	820 µg/mL
Content MPLA	84 µg/mL
Content cholesterol	196 µg/mL
Content DPPC	93 µg/mL
Particle size (z-average)	49 nm
Polydispersity index (PDI)	0.15
Residual MEGA-10	< 1 µg/mL
pH	6.5
Osmolality	281 mOsmol/kg
Endotoxin	< 1 EU/mL
Bioburden	0 CFU / 10 mL

Supplemental Table 2. Dilution steps for SMNP GMP-process synthesis

Dilution step	Dilution factor	Hold time between adding steps
1	10x	10 min
2	25x	45 min
3	30x	20 min
4	35x	20 min
5	40x	10 min
6	45x	10 min
7	50x	10 min
8	60x	10 min
9	75x	10 min
10	85x	10 min
11	100x	10 min

Supplemental Table 3. Staining panel for antigen-specific B_{Mem} in PBMCs

Reagent	Clone	Source (Catalog #)	Dilution
BV421 Streptavidin	-	BioLegend (405225)	-
AF647 Streptavidin	-	BioLegend (405237)	-
PE Streptavidin	-	BioLegend (405245)	-
Viability APC-eFluor780	-	Thermo Fisher Scientific (65-0865-14)	1:2000
CD8 BV510	RPA-T8	BD Biosciences (563256)	1:100
CD16 BV510	3G8	BioLegend (302048)	1:100
CD14 BV510	M5E2	BioLegend (301842)	1:100
CD3 BV510	SP34-2	BD Biosciences (740187)	1:100
CD20 BUV395	2H7	BD Biosciences (563781)	1:100
IgG BUV737	G18-145	BD Biosciences (612819)	1:100
CD21 BV711	B-ly4	BD Biosciences (563163)	1:50
CD27 PE-Cy7	O323	Thermo Fisher Scientific (25-0279-42)	1:50
IgM PerCP-Cy5.5	G20-127	BD Biosciences (561285)	1:50
IgD AF488	Polyclonal	Southern Biotech (2030-30)	1:50

Supplemental Table 4. Staining panel for antigen-specific B_{GC} and B_{Mem} in ipsilateral LN FNAs

Reagent	Clone	Source (Catalog #)	Dilution
BV421 Streptavidin	-	BioLegend (405225)	-
AF647 Streptavidin	-	BioLegend (405237)	-
PE Streptavidin	-	BioLegend (405245)	-
Viability eFluor506	-	Thermo Fisher Scientific (65-0866-18)	1:1000
CD4 BV711	OKT4	BioLegend (317440)	1:100
CD8 APC-eFluor780	RPA-T8	Thermo Fisher Scientific (47-0088-42)	1:100
CD16 APC-eFluor780	CB16	Thermo Fisher Scientific (47-0168-42)	1:100
CD20 AF488	2H7	BioLegend (302316)	1:100
CD38 PE-Cy5	OKT10	Conjugated in house (see Methods)	1:100
IgG BUV737	G18-145	BD Biosciences (612819)	1:100
CD3 BUV395	SP34-2	BD Biosciences (564117)	1:40
IgM PerCp-Cy5.5	G20-127	BD Biosciences (561285)	1:40
PD-1 BV605	EH12.2H7	BioLegend (329924)	1:40
CXCR5 PE-Cy7	Mu5UBEE	Thermo Fisher Scientific (25-9185-42)	1:20
CD71 PE-CF594	L01.1	BD Biosciences (Custom, see Methods)	1:20

Supplemental Table 5. Staining panel for antigen-specific B_{GC} and B_{Mem} in contralateral LN FNAs

Reagent	Clone	Source (Catalog #)	Dilution
BV421 Streptavidin	-	BioLegend (405225)	-
AF647 Streptavidin	-	BioLegend (405237)	-
PE Streptavidin	-	BioLegend (405245)	-
Viability APC-eFluor780	-	Thermo Fisher Scientific (65-0865-14)	1:2000
CD8 BV510	RPA-T8	BD Biosciences (563256)	1:100
CD16 BV510	3G8	BioLegend (302048)	1:100
CD14 BV510	M5E2	BioLegend (301842)	1:100
CD3 BV510	SP34-2	BD Biosciences (569486)	1:100
CD20 BUV395	2H7	BD Biosciences (563782)	1:100
IgG BUV737	G18-145	BD Biosciences (612819)	1:100
CD38 PE-Cy5	OKT10	Conjugated in house (see Methods)	1:200
CD27 PE-Cy7	O323	Thermo Fisher Scientific (25-0279-42)	1:50
CD21 BV711	B-ly4	BD Biosciences (563163)	1:50
IgM PerCP-Cy5.5	G20-127	BD Biosciences (561285)	1:50
IgD AF488	Polyclonal	Southern Biotech (2030-30)	1:50
CD69 BV650	FN50	BD Biosciences (563835)	1:100
CD71 PE-CF594	L01.1	BD Biosciences (Custom, see Methods)	1:20

Supplemental Table 6. Staining panel for sorting antigen-specific B_{Mem} in PBMCs

Reagent	Clone	Source (Catalog #)	Dilution
TotalSeq-C BV421 Streptavidin	-	BioLegend (Custom, see Methods)	-
AF647 Streptavidin	-	BioLegend (405237)	-
TotalSeq-C PE Streptavidin	-	BioLegend (405261)	-
Viability APC-eFluor780	-	Thermo Fisher Scientific (65-0865-14)	1:2000
CD8 BV510	RPA-T8	BD Biosciences (563256)	1:100
CD16 BV510	3G8	BioLegend (302048)	1:100
CD14 BV510	M5E2	BioLegend (301842)	1:100
CD3 BV510	SP34-2	BD Biosciences (569486)	1:100
CD20 BUV395	2H7	BD Biosciences (563782)	1:100
IgG BV605	G18-145	BD Biosciences (563246)	1:100
IgM PerCP-Cy5.5	G20-127	BD Biosciences (561285)	1:50
IgD AF488	Polyclonal	Southern Biotech (2030-30)	1:50
CD27 PE-Cy7	O323	Thermo Fisher Scientific (25-0279-42)	1:50

Supplemental Table 7. AIM/ICS staining panel for antigen-specific T cells in PBMCs

Reagent	Clone	Source (Catalog #)	Dilution
LIVE/DEAD Fixable Blue	-	Thermo Fisher Scientific (L23105)	1:500
CXCR5 PE-Cy7	MU5UBEE	Thermo Fisher Scientific (25-9185-42)	1:100
CCR7 BV650	G043H7	BioLegend (353233)	1:100
CD69 PE-Cy5	FN50	BioLegend (310908)	1:250
CD137 (4-1BB) BV421	4B4-1	BioLegend (309819)	1:250
CD25 BV605	BC96	BioLegend (302631)	1:250
CD40L BB515	24-31	BD Biosciences (568170)	1:250
CD134 (OX40) PE	L106	BD Biosciences (340420)	1:250
CD8 BUV496	RPA-T8	BD Biosciences (612943)	1:100
CD14 APC-Cy7	M5E2	BioLegend (301820)	1:100
CD16 APC-eFluor780	eBioCB16	Thermo Fisher Scientific (47-0168-42)	1:100
CD20 APC-Cy7	2H7	BioLegend (302314)	1:100
CD3 BUV395	SP34-2	BD Biosciences (564117)	1:100
CD4 PerCP-Cy5.5	OKT4	BioLegend (317428)	1:100
PD-1 BV785	EH12.2H7	BioLegend (329929)	1:100
CD45RA PE-CF594	5H9	BD Biosciences (565419)	1:100
ICOS BV480	C398.4A	BD Biosciences (566087)	1:100
IFN-γ BV750	B27	BD Biosciences (566357)	1:100
IL-2 BUV737	MQ1-17H12	BD Biosciences (612836)	1:200
TNF BV711	MAb11	BioLegend (502940)	1:200
Granzyme B Alexa Fluor 700	GB11	BD Biosciences (560213)	1:1000
IL-21 Alexa Fluor 647	3A3-N2.1	BD Biosciences (560493)	1:200
